# Endogenous/exogenous dual-responsive nanozyme for photothermally enhanced ferroptosis-immune reciprocal synergistic tumor therapy

**DOI:** 10.1126/sciadv.adq3870

**Published:** 2025-05-14

**Authors:** Hanxi Zhang, Jiazhen Lv, Hao Wu, Yuhan He, Mengyue Li, Chunhui Wu, Dong Lv, Yiyao Liu, Hong Yang

**Affiliations:** ^1^ Department of Oncology & Cancer Institute, Sichuan Provincial People’s Hospital, and School of Life Science and Technology, University of Electronic Science and Technology of China, Chengdu 610054, Sichuan, P. R. China.; ^2^ TCM Regulating Metabolic Diseases Key Laboratory of Sichuan Province, Hospital of Chengdu University of Traditional Chinese Medicine, No. 39 Shi-er-qiao Road, Chengdu 610072, Sichuan, P. R. China.; ^3^ Department of Urology, Deyang People’s Hospital, Deyang 618099, Sichuan, P. R. China.

## Abstract

Apoptosis resistance and immune evasion of tumor cells substantially increase the risk of cancer treatment failure. Here, a multifunctional nanozyme MET-CMS@FeTA (MCMSFT) formulated to induce nonapoptotic ferroptosis and boost immune recognition/attack, where compensatory mechanisms collectively overcome intrinsic tumor therapeutic limitations and improve medical intervention outcomes. Leveraging the multienzyme-like activity of MCMSFT to achieve oxygen generation, hydroxyl radical production, and glutathione depletion promotes hypoxia relief and triggers apoptosis/ferroptosis. Notably, MCMSFT-mediated photothermal therapy (PTT) facilitates direct tumor thermal ablation and offers exogenous heat to accelerate nanocatalytic reactions. Furthermore, PTT/ferroptosis-caused immunogenic cell death favors antitumor immunity initiation. Simultaneously, metformin administration and hypoxia amelioration down-regulate programmed death ligand 1 alleviating immune evasion. Interferon-γ secretion poses positive feedback to ferroptosis, thereby establishing a ferroptosis-immune mutual amplification loop. Antitumor performances illustrate that MCMSFT eliminates primary tumors and suppresses metastasis/rechallenge tumors. Collectively, MCMSFT surmounts the predicament of apoptosis resistance and immune evasion in cancer treatment to acquire more effective and comprehensive therapy efficacy.

## Teaser

Responsive nanozyme-mediated multimodal/multipathway/multilevel reciprocal synergistic tumor therapy induces hybrid cell death.

## INTRODUCTION

The tumor microenvironment (TME) is typically characterized by slight acidity, hypoxia, and excessive levels of hydrogen peroxide (H_2_O_2_)/glutathione (GSH), attributable to rapid cell proliferation, abnormal metabolism, and inadequate blood supply (*1*–*3*). These alterations create challenges for conventional cancer treatments yet provide opportunities for TME stimuli-responsive nanoformulation applications (*4*, *5*). Nanozymes refer to nanomaterials with intrinsic enzyme-mimicking catalytic activities, which harness the pathological features of TME to trigger relevant chemical reactions in situ to launch tumor nanocatalysis therapy (NCT) (*6*–*9*). With the advent of nanozyme application in tumor NCT, various nanozymes have been developed and proven desirable candidates for cancer treatment. In line with enzyme-like activity, nanozymes can roughly divide into the following categories: those mimicking peroxidase (POD), catalase (CAT), oxidase, superoxide dismutase, and glutathione peroxidase (GPx) (*10*–*12*). The active sites of nanozymes generally are metal components, and metallic elements can effectively simulate the electronic redox processes catalyzed by natural enzymes, which endow nanozymes with the advantages of sustained, controllable, and multiplex enzyme activities (*6*, *7*, *13*–*15*). Therefore, rationally designed nanozymes can integrate diverse therapeutic approaches to inducing multiple forms of cell death for effective and specific eradication of tumor cells. For instance, POD-like nanozymes can catalyze tumor endogenous H_2_O_2_ to produce cytotoxic hydroxyl radicals (·OH) to kill tumor cells (*9*, *16*, *17*). The increase in intracellular ·OH, in addition to inducing apoptosis, can elicit ferroptosis by peroxidizing membrane lipids and impairing antioxidant defenses (*18*–*23*). Ferroptosis is a type of oxidative nonapoptotic cell death driven by glutathione peroxidase 4 (GPX4) inactivation and lipid peroxide (LPO) overwhelming accumulation, which provides a complementary solution to address apoptosis resistance in cancer treatment (*24*, *25*).

Although tumor NCT demonstrates respectable prospects in cancer treatment, its therapeutic efficacy is severely compromised by the inherent drawback of restricted reaction activity due to unfavorable catalytic conditions in the tumor milieu (*20*–*22*, *26*). Thus, nanocatalytic efficiency enhancement and combinational therapy strategy adoption are demanded for pursuing more effective treatment outcomes. The Arrhenius equation suggests that it is feasible to speed the reaction rate by increasing the temperature, reflecting that photothermal therapy (PTT) can serve as an ideal supportive manner to provide an exogenous driving force for accelerating catalytic reactions in addition to direct tumor thermal ablation (*22*, *27*). Moreover, compared with the single metal–based nanozyme, nanozyme containing bimetallic/multiple metal elements illustrated higher catalytic activity owing to the synergistic effect of different metals (*11*). Consequently, multiple-metal nanozymes with excellent photothermal conversion capacity hold promise for surmounting the obstacle of limited catalytic activity. Furthermore, growing evidence reveals that PTT and ferroptosis occur accompanied by immunogenic cell death (ICD), inducing the release of damage-associated molecular patterns (DAMPs) and tumor-associated antigens (TAAs) (*21*, *28*). These stimulators are crucial to activate antitumor immune responses, which remove residual/metastatic tumor cells and prevent tumor relapse after therapeutic intervention withdrawal. Nevertheless, cancer cells tend to evoke immunosuppressive mechanisms of programmed death ligand 1 (PD-L1) up-regulation to evade immune recognition and attack, causing T lymphocyte dysfunction and exhaustion (*29*–*31*). Metformin (MET), a safe, effective, and economical substitute for PD-L1 antibody, has been reported to down-regulate PD-L1 expression to restore antitumor immunity of T lymphocytes (*32*, *33*). Recent studies have highlighted that raised interferon-γ (IFN-γ) secretion stemming from immune activation exacerbates ferroptosis (*21*, *34*–*36*). Hence, we reasonably speculate that tactfully using the subtle reinforcement among PTT, nanocatalysis, and immunomodulation may achieve optimized tumor therapeutic efficacy.

**Fig. 1.**
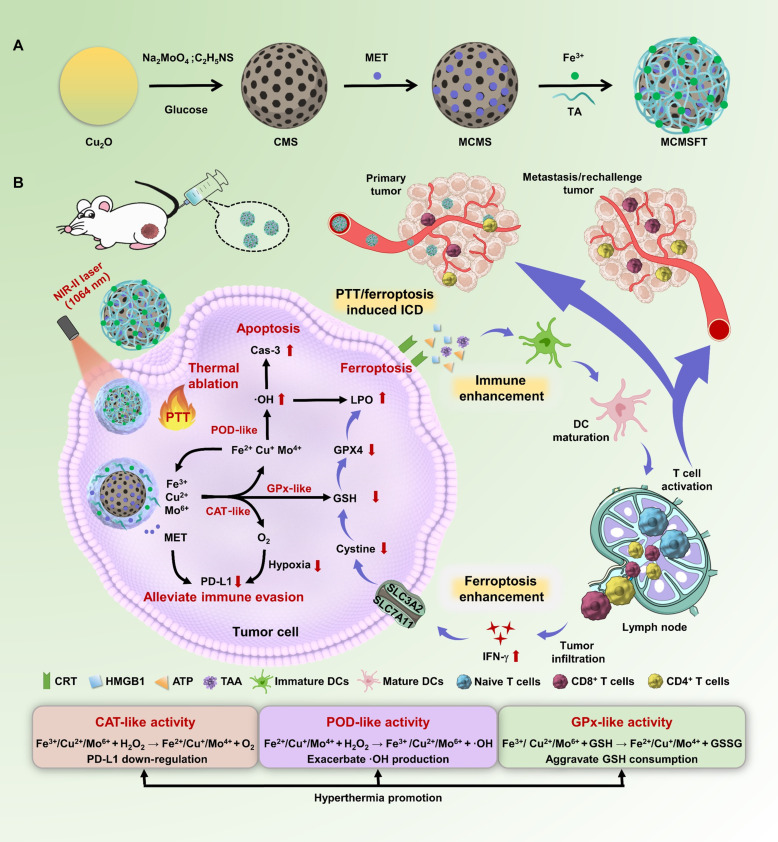
Schematic illustration of the multifunctional nanozyme MCMSFT for endogenous/exogenous dual-responsive cancer treatment. (**A**) The fabrication procedure of MCMSFT. (**B**) The underlying mechanism of MCMSFT-mediated photothermally enhanced nanocatalysis and immunomodulation reciprocal synergistic therapy induced hybrid cell deaths of apoptosis/ferroptosis/necroptosis in breast cancer. GSSG, oxidized glutathione.

In this work, an endogenous/exogenous dual-responsive multifunctional nanozyme MET-CMS@FeTA (MCMSFT) was developed for photothermally enhanced nanocatalysis and immunomodulation reciprocal synergistic therapy against tumors (Fig. 1). The hollow mesoporous copper molybdenum sulfide (Cu_2_MoS_4_; CMS) was used as a core platform to encapsulate the immune adjuvant MET and then coated with metal-polyphenol networks (MPNs) spontaneously formed by ferric iron (Fe^3+^) and tannic acid (TA), yielding MCMSFT. MCMSFT exhibits tumor-specific catalytic properties and thermally promoted multiple enzyme-like activities, which are competitive in high-efficiency tumor NCT. Intracellular H_2_O_2_/GSH and laser irradiation worked as endogenous and exogenous stimuli, respectively, to initiate and accelerate the catalytic reaction at tumor sites. The triple enzyme-like activity of CAT, POD, and GPx had by MCMSFT could achieve multiple goals for supplying oxygen, producing ·OH, and depleting GSH contemporaneously through cycling valence state alteration of metal ions. Rapid ·OH generation and substantial GSH consumption provoked tumor cell apoptosis and ferroptosis. Meanwhile, tumor thermal ablation and ferroptosis triggered ICD to stimulate dendritic cells (DCs) maturation and T cell activation. Parallelly, PD-L1 down-regulation due to MET addition and hypoxia mitigation relieved immune evasion, thereby sensitizing immunosurveillance to facilitate the recognition and attack of tumor cells. Impressively, immune activation induced increased IFN-γ secretion, inhibiting the expression of solute carrier family 3 member 2 (SLC3A2) and solute carrier family 7 member 11 (SLC7A11), and further amplified ferroptosis by arresting GSH synthesis. Overall, our results demonstrate that the elaborately designed MCMSFT effectively integrates multimodal treatment approaches, multipathway therapeutic mechanisms, and multilevel intervention strategies, to eradicate tumor lesions via apoptosis/ferroptosis/necroptosis hybrid cell death. The seamless synergism of PTT, NCT, and immunomodulation provides a high-performance anticancer paradigm that overcomes apoptosis resistance and immune evasion, promoting tumor remission and preventing metastasis or reoccurrence.

## RESULTS

### Preparation and characterization of MCMSFT

**Fig. 2.**
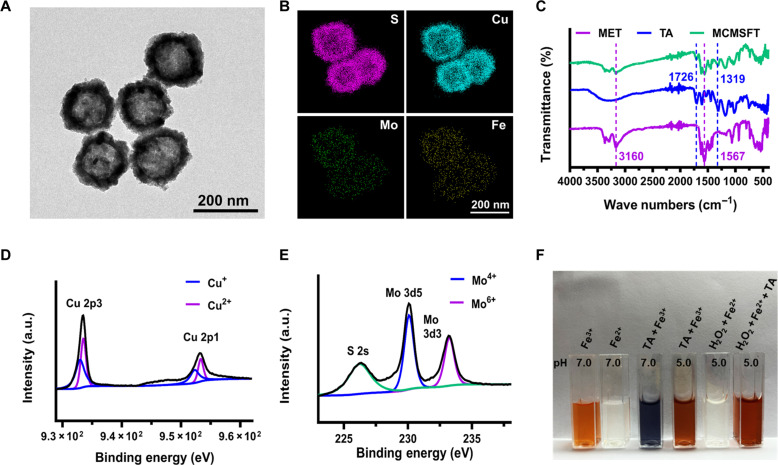
Characterization of MCMSFT. (**A**) TEM image of MCMSFT. (**B**) STEM-EDX element mapping of MCMSFT. (**C**) The FTIR spectra of MET, TA, and MCMSFT. High-resolution (**D**) Cu 2p and (**E**) Mo 3d XPS spectra of MCMSFTs. a.u., arbitrary units. (**F**) Color reaction observation for valence changes of iron ions using *o*-phenanthroline as an indicator. The *o*-phenanthroline reacts with Fe^2+^ to produce an orange complex, while the complex formed with Fe^3+^ is colorless. Fe^3+^ chelate with TA to form stable blue complexes under neutral conditions.

Hollow mesoporous CMS was obtained following the solvothermal method described in previous studies (*37*, *38*). Transmission electron microscopy (TEM) images (fig. S1, A and B) revealed that the sacrificial template Cu_2_O exhibited a solid spherical morphology, while CMS formed a hollow structure with a large cavity. The porous structure of CMS was confirmed by nitrogen adsorption and desorption analysis, showing an average pore size of ~4 nm (fig. S1, C and D). The x-ray diffraction (XRD) pattern displayed the characteristic diffraction peaks of CMS (fig. S1E), which is consistent with referenced literature (*38*). The x-ray photoelectron spectroscopy (XPS) survey indicated the presence of S, Cu, and Mo elements in CMS (fig. S1F). Subsequently, MCMSFT was obtained through sequential MET loading and surface modification with MPNs. The drug loading investigation (fig. S2A) suggested that the optimal feeding mass ratio of MET to CMS was 1:1 and the encapsulation efficiency and drug loading of MET were 83.2 and 45.9%, respectively. As shown in Fig. 2A, the analogous morphology and structure to CMS were observed in the TEM image of MCMSFT, with an average hydrodynamic diameter of MCMSFT of 169.7 ± 5.8 nm and a zeta potential of −14.9 ± 0.46 mV in the phosphate-buffered saline (PBS) buffer (fig. S2, B and C). In addition, MCMSFT witnessed negligible size and protein disulfide isomerase alterations in the PBS buffer within a week, indicating that MCMSFT have relatively high stability and dispersity in physiological solution (fig. S2D). Scanning TEM (STEM)–energy-dispersive x-ray (EDX) element mapping images (Fig. 2B) illustrated the coexistence and homogeneous distribution of S, Cu, Mo, and Fe in MCMSFT. Surface modification and drug encapsulation were confirmed according to the Fourier transform infrared (FTIR) spectra of MCMSFT (Fig. 2C), which witnessed the characteristic peaks at 3160 and 1567 cm^−1^ attributed to N─H stretching and C─N stretching for MET, whereas the characteristic peaks at 1726 and 1319 cm^−1^ ascribed to the carboxyl carbonyl group and the phenol group of TA. These results provided credible evidence for the successful fabrication of MCMSFT.

Mechanistically, nanoenzymes simulate the function of natural enzymes using multivalent metal ions as redox couples to actuate catalytic reactions (*8*, *39*). To explore the potential of MCMSFT as nanozymes for biomedical applications, we adopted high-resolution XPS spectroscopy to analyze the valence states of metal ions in MCMSFT. As shown in Fig. 2 (D and E), the XPS spectra of MCMSFT in Cu 2p and Mo 3d regions uncovered that Cu is composed of Cu^+^ (932.98 eV, 952.28 eV)/Cu^2+^ (933.48 eV, 953.38 eV) and Mo was presented in the valence state of Mo^4+^ (230.08.2 eV)/Mo^6+^ (233.28.5 eV). The dynamic coordination of ferric irons and TA constructed one of the simplest MPNs, with pH-responsive disassembly and biocompatible improvement properties (*40*–*42*). The iron ions initially chelated on the surface of MCMSFT were exclusively in the form of Fe^3+^. In the TME, acid-active reduction of TA and excessive H_2_O_2_ triggered Fenton reaction, making an additional redox couple of Fe^2+^/Fe^3+^ seem acquirable. As photograph visualized in Fig. 2F, under acidic conditions, MPN depolymerization dissociated TA, inducing the release of Fe^3+^, which was transduced to Fe^2+^. In the presence of H_2_O_2_, Fe^2+^ participated in the Fenton reaction turn into Fe^3+^, and with TA addition, Fe^3+^ produced by the Fenton reaction is regenerated as Fe^2+^. These findings validated the existence of multivalent metal ions in MCMSFT, which act as redox couples and lay the foundation for MCMSFT to have enzyme-mimicking activity for tumor NCT.

### Photothermal performance and multienzyme-like activity

**Fig. 3.**
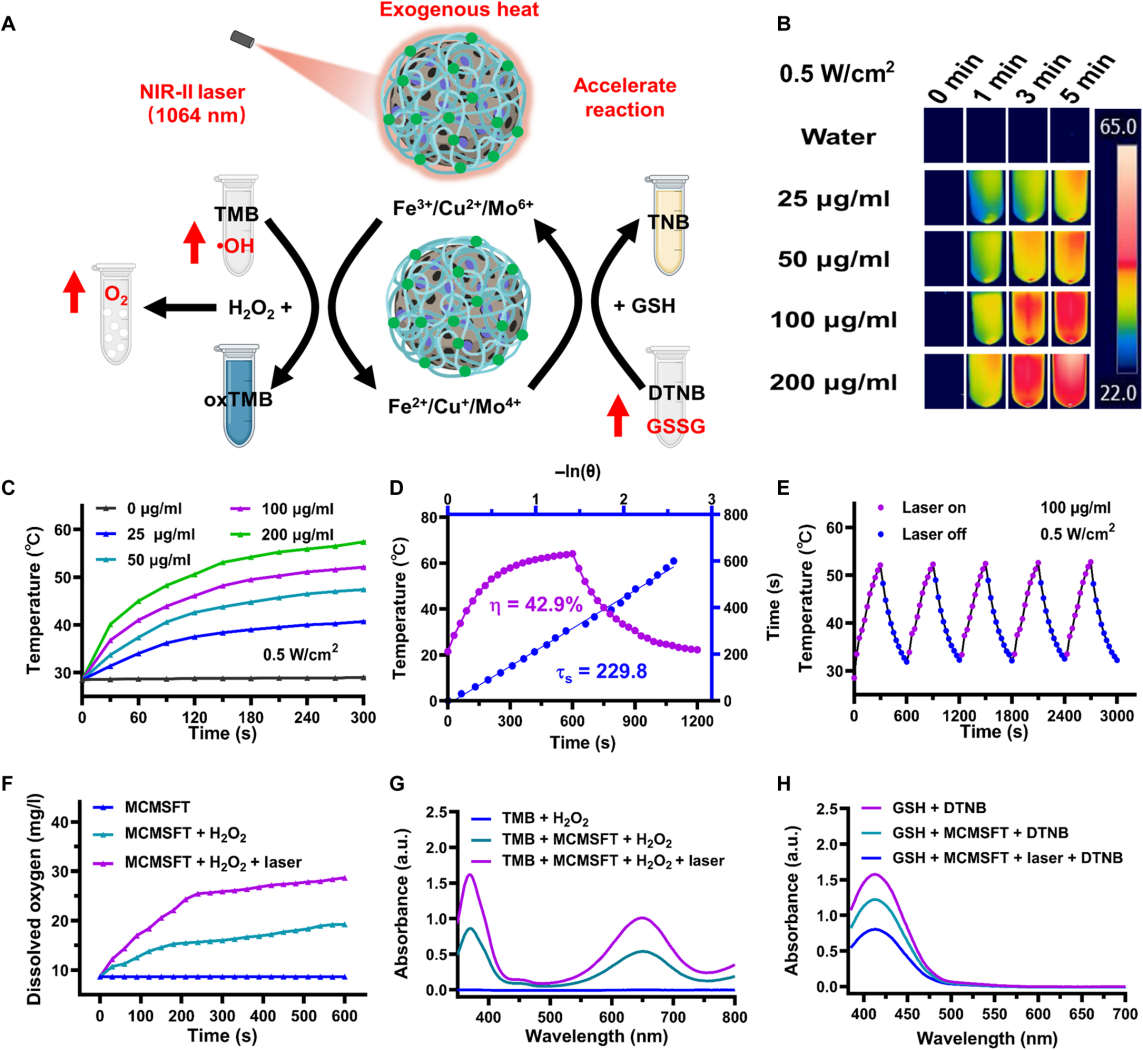
Photothermal performance and multienzyme-like activity. (**A**) Schematic illustration of laser irradiation-induced temperature increase accelerates catalytic reaction to boost multienzyme-like activities of MCMSFT. (**B**) Infrared thermal images and (**C**) photothermal heating curves of various concentrations of MCMSFT with laser irradiation. (**D**) Linear time data of heating and cooling period and the associated time constant for heat transfer of MCMSFT obtained from the cooling stage. (**E**) Recycling-heating profiles of MCMSFT for five laser on/off cycles. (**F**) Dissolved oxygen curves of MCMSFT with various treatments. H_2_O_2_, 100 μM; laser irradiation: 1064 nm, 0.5 W/cm^2^, 5 min. (**G**) The absorption spectra of the ·OH production exanimation with tetramethyl-benzidine (TMB) (1 mM) as the probe. (**H**) The absorption spectra of the GSH (10 mM) depletion detection with 5,5-dithiobis (2-nitrobenzoic acid) (DTNB) (5 mM) as the probe.

Given that MCMSFT harbored multivalent elements for nanocatalytic reactions and observed a strong absorption in the NIR-II bio-window (fig. S3A), MCMSFT embraced both photothermal and enzymatic functions in a single platform to became a competitive multifunctional nanozyme (Fig. 3A). To evaluate the photothermal transduction capability of MCMSFT, we monitored and photographed the temperature variations of samples under 1064-nm laser irradiation. The infrared thermal images (Fig. 3B) and photothermal heating curves (Fig. 3C and fig. S3B) demonstrate that the temperatures elevated gradually as the concentration of the MCMSFT increased, the irradiation time prolonged, and the laser power density enlarged. Notably, rapid temperature increase and suitable temperature maintenance were acquired when MCMSFT (100 μg/ml) was subjected to laser irradiation (0.5 W/cm^2^) for 5 min. In this situation, relatively high photothermal temperatures were obtained to effectively kill tumor cells while avoiding normal tissue damage from overlong hyperthermia, which can be considered a suitable dose of MCMSFT for subsequent experiments. Furthermore, to quantitatively assess the photothermal conversion capacity of MCMSFT, we calculated photothermal conversion efficiency (η) to be ~42.9% using data during the cooling stage (Fig. 3D). Meanwhile, to investigate the photothermal stability of MCMSFT, we repeated five times the heating-cooling cycle. MCMSFT exhibited nearly unchanged temperature elevation, indicating the prominent photostabilities of MCMSFT (Fig. 3E). These results proved that MCMSFT had a remarkable and stable photothermal effect to induce temperature increase with massive potential for tumor hyperthermia therapy.

The enzyme-like activity of MCMSFT was studied in a buffer that emulates the conditions of lysosomes or endosomes (pH 5.0; 10 mM GSH and 100 μΜ H_2_O_2_), where the surface coating of MCMSFT depolymerizes rapidly, exposing the nanocatalyst to initiate nanocatalytic reactions. To assess the CAT-like activity of MCMSFT, we detected its capability to decompose H_2_O_2_ into oxygen. The increased dissolved oxygen and the appearance of numerous bubbles signify substantial oxygen generation, which is further accelerated and enhanced with laser irradiation (Fig. 3F and fig. S4A). Similarly, to evaluate the POD-like activity of MCMSFT, we measured its ability to induce ·OH production. Tetramethyl-benzidine (TMB) was used as a probe, reacting with ·OH to produce blue oxidized TMB, characterized by a distinctive absorption peak at 652 nm (*20*, *21*, *38*). As shown in Fig. 3G and fig. S4B, obvious characteristic absorption and a blue color change of the sample solution indicated efficient ·OH production. More intensive absorption evidenced a higher level of ·OH formation upon laser irradiation. Likewise, to investigate the GPx-like activity of MCMSFT, we examined its competence in depleting GSH. 5,5-Dithiobis (2-nitrobenzoic acid) (DTNB) was adopted as an indicator, which react with GSH to form yellow-colored 5-thio-2-nitrobenzoic acid (TNB), exhibiting a typical absorbance at 412 nm (*20*, *21*). The marked decrease in absorbance and the faded color of the sample solution suggest obvious GSH reduction. Further absorbance decline was observed once laser irradiation was implemented indicating more vigorous GSH consumption (Fig. 3H and fig. S4C). These observations revealed that MCMSFT exhibited attractive photothermally enhanced CAT/POD/GPx-like multienzyme activity, admitted as multifunctional nanozyme for application in tumor NCT.

### Dual-responsive drug release and cellular uptake

The TME-stimulative nanoformulations significantly enhance drug enrichment and cytoplasmic release at the tumor site, substantially maximizing therapeutic intervention efficacy and lowering the adverse effects (*43*, *44*). The release profiles of MET were studied in PBS buffers mimicking physiological conditions (pH 7.4) and tumor milieu conditions (pH 6.5 at TME or pH 5.0 at lysosomes with 10 mM GSH and 100 μM H_2_O_2_). In fig. S5, the cumulative release curves of MET displayed that acidity decline and laser irradiation increased MET release due to acid-sensitive dissociation of surface coating and the photothermally promoted molecular diffusion, which accelerated drug liberation. In addition, the possible biodegradation of MCMSFT was visually investigated by TEM (fig. S6). The integrity shape of MCMSFT was maintained under physiological conditions. Inversely, MCMSFT witnessed a severe structure collapse after 72 hours of incubation in the tumor milieu mimicking buffers, and the disintegration was more thorough under lysosomal conditions. Therefore, MCMSFT was capable of maintaining stability during blood circulation but quickly collapsed to release drugs at tumor sites. These findings suggest that MCMSFT can achieve controlled drug release and precise tumor therapy in response to endogenous acidic conditions and exogenous laser irradiation stimulation.

**Fig. 4.**
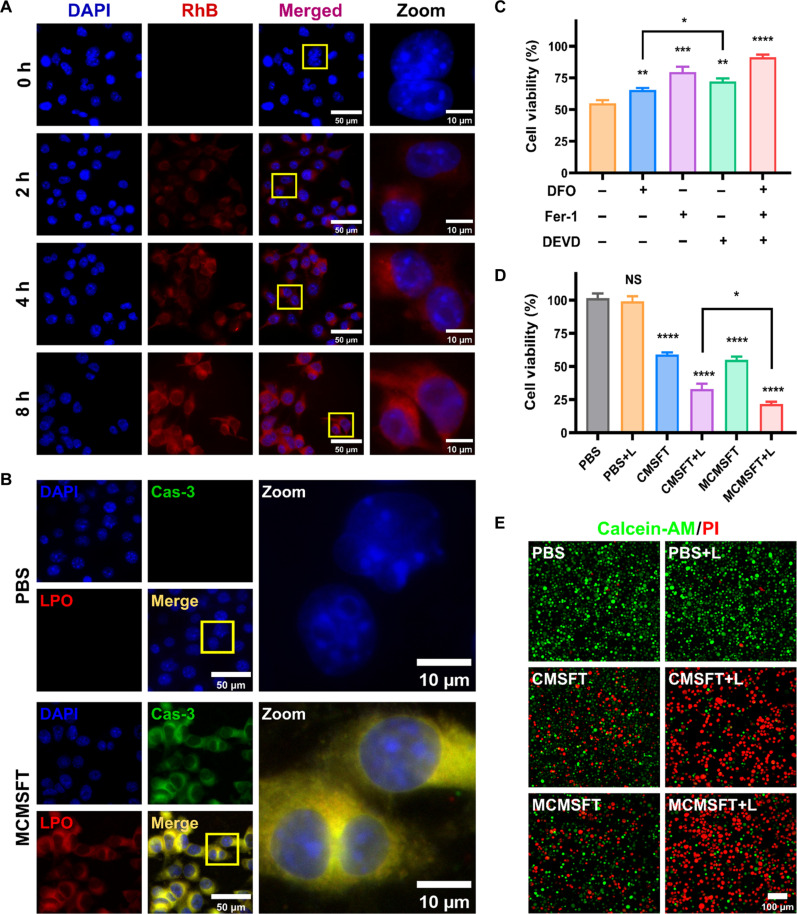
Cellular uptake and in vitro therapeutic effect. (**A**) Fluorescence images of 4T1 cells incubated with RhB-MCMSFT for various time intervals. h, hours. (**B**) Fluorescence images of caspase-3 (Cas-3) immunofluorescence staining and LPO staining in 4T1 cells after incubation with PBS or MCMSFT. (**C**) The cell viability of 4T1 cells treated with MCMSFT in the presence of DFO (100 μM), Fer-1(10 μM), or DEVD (50 μM). (**D**) The cell viability and (**E**) live/dead cell staining images of 4T1 cells treated with different formulations with or without laser irradiation. The data are presented as the means ± SD, *n* = 3, *P < 0.05, **P < 0.01, ***P < 0.001, and ****P < 0.0001. NS, no significance difference. AM, acetoxymethyl ester.

Given that acceptable biocompatibility is a prerequisite for advancing nanomaterials applications (*45*, *46*). To investigate the biocompatibility of MCMSFT, we measured the cell viability of NIH3T3 normal cells and 4T1 or EMT-6 tumor cells after incubation with various concentrations of MCMSFT. As shown in fig. S7, NIH3T3 cells maintained high cell viability even when treated with high concentrations of MCMSFT up to 200 μg/ml. However, the cell viability of 4T1 or EMT-6 tumor cells substantially decreased, as the concentration of MCMSFT reached 100 μg/ml. These results suggest that MCMSFT does not significantly affect the cell viability of normal cells while exhibiting tumor-specific cytotoxicity. More severe cytotoxicity in the 4T1 cell line is attributed to its higher levels of intracellular H_2_O_2_ (*22*). Subsequently, the fluorescence-labeled rhodamine B (RhB)–MCMSFT was prepared to investigate the cellular uptake behavior in 4T1 cells. Fluorescence images presented that the distribution of red fluorescence within the cells became brighter and more extensive with increased incubation time (Fig. 4A). Flow cytometry quantification also confirmed the increase in fluorescence signal intensity, indicating a time-dependent continuous internalization of RhB-CMSFT (fig. S8). Moreover, the subcellular distribution study exposed that RhB-CMSFT and lysosomes exhibited obvious colocalization, meaning that MCMSFT primarily localized in lysosomes after entering cells (fig. S9). This subcellular localization is conceptually desirable since the acidic condition of lysosomes (pH 4.5 to 5.5) facilitates MCMSFT dissociation and promotes therapeutic agent liberation and exposure.

### In vitro therapeutic efficacy and potential mechanism

Caspase-3 (Cas-3) protein and LPO serve as important biomarkers of apoptosis and ferroptosis, respectively (*21*, *22*). To assess the feasibility of MCMSFT overcoming apoptosis resistance, we examined cell death forms by Cas-3 immunofluorescent staining and LPO fluorescent staining after MCMSFT treatment. The green fluorescence of Cas-3 indicated cellular apoptosis occurrence, while the red fluorescence of LPO suggested ferroptosis existence (Fig. 4B). Typical ferroptosis- and apoptosis-related inhibitors were used to study their influences on cell activity after MCMSFT treatment. As displayed in Fig. 4C, ferrostatin-1 (Fer-1; a typical ferroptosis inhibitor), deferoxamine (DFO; a specific iron chelating agent), and Acetyl-Asp-Glu-Val-Asp-aldehydeAc-DEVD-CHO (DEVD; a typical apoptosis inhibitor) all rescued cells from MCMSFT-induced cell death, confirming that MCMSFT triggered apoptosis and ferroptosis in tumor cells contemporaneously. Thereafter, to explore the therapeutic efficacy of different medical interventions, we determined the cell viability after various treatments (Fig. 4D). Absent of laser irradiation, CMSFT or MCMSFT caused moderate cell death due to their catalytic activity. However, the cell viability displayed an appreciable drop once the cells were treated with CMSFT or MCMSFT plus laser irradiation (CMSFT+L or MCMSFT+L, respectively). Specifically, lower cell viability in the MCMSFT+L group than in the CMSFT+L group may attribute to MET inhibiting GPX4 expression to promote ferroptosis (*47*, *48*). Correspondingly, the live and dead cell staining assay further authorized that MCMSFT-mediated photothermally enhanced synergistic therapy obtained the highest cytotoxicity among all treatments (Fig. 4E). These data convincingly demonstrated that MCMSFT was capable of inducing apoptosis and ferroptosis coincidently and the photothermal effect of MCMSFT has irreplaceable advantages in exacerbating hybrid tumor cell death.

**Fig. 5.**
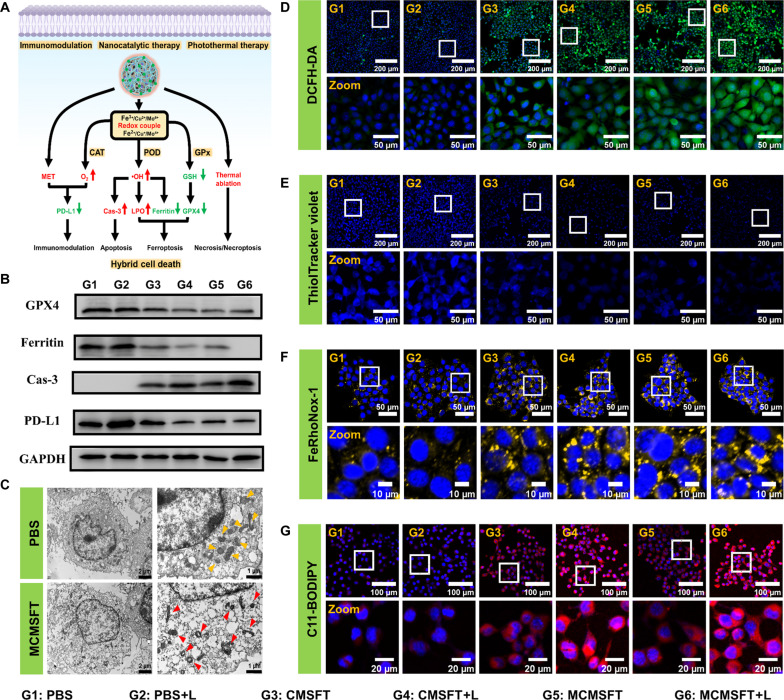
Potential mechanism of hybrid multiple cell deaths induction. (**A**) The proposed molecular mechanisms of MCMSFT-induced hybrid cell death including apoptosis, ferroptosis, and necroptosis. (**B**) Representative Western blot images of GPX4, ferritin, Cas-3, and PD-L1 expression measurement after different treatments. GAPDH, glyceraldehyde-3-phosphate dehydrogenase. (**C**) Bio-TEM images of 4T1 cells treated with PBS or MCMSFT. (**D** to **G**) Fluorescence staining images of intracellular Fe^2+^, ROS, GSH, and LPO detection in 4T1 cells after different treatments.

The favorable in vitro antitumor efficacy of MCMSFT encouraged further investigation to explore the possible therapeutic molecular mechanisms. Potential regulatory molecules that induce hybrid cell death by MCMSFT-mediated PTT/nanocatalysis/immunomodulation synergistic therapy were summarized in Fig. 5A, and a series of experiments were conducted to validate the proposed mechanism. Hypoxia has been reported to promote the activation of the transcriptional factor hypoxia-inducible factor–1α (HIF-1α) and then induce the up-regulation of PD-L1 expression to reinforce immunosuppression (*49*, *50*). To evaluate the cellular hypoxia–relief capability of MCMSFT, we measured the intracellular oxygen generation of hypoxic cultured 4T1 cells after MCMSFT treatment using tris(4,7-diphenyl-1,10-phenanthroline)ruthenium(II) dichloride {[Ru(dpp)_3_]Cl_2_} as an oxygen sensor. Compared to the bright red fluorescence observed in the PBS group, remarkable fluorescence quenching occurred in the MCMSFT group, demonstrating that the MCMSFT group provided substantial oxygen effectively alleviating cellular hypoxia (fig. S10). Further, Western blotting analysis showed the most down-regulated PD-L1 expression in the MCMSFT+L group, pointing a more prominent reversal of tumor immunosuppression due to MET administration and oxygen supply (Fig. 5B and fig. S11A). Both ferroptosis and apoptosis are accompanied by mitochondrial dysfunction, manifested by mitochondrial shrinkage and the loss of mitochondrial membrane potential (MMP) (*20*, *21*). The morphological changes of the mitochondria before and after MCMSFT treatment were visualized by bio-TEM. As depicted in Fig. 5C, compared with the PBS group, reduced mitochondrial volume and increased membrane density were observed after MCMSFT treatment, verifying that the MCMSFT facilitated ferroptosis and apoptosis of tumor cells. In addition, mitochondrial dysfunction was examined using JC-1 staining, reflecting the loss of MMP. Cells treated with MCMSFT+L exhibited the most vigorous green and the weakest red fluorescence (fig. S12), suggesting that the most severe mitochondrial damage in the MCMSFT+L group is probably attributed to enhanced apoptosis and ferroptosis.

Reactive oxygen species (ROS) dominated by ·OH are regarded as the chief culprit capable of simultaneously inducing tumor cell apoptosis and ferroptosis (*22*, *23*). Intracellular ROS level was evaluated by 2,7-dichlorofluorescein (DCFH) assay after various treatments. Obvious green fluorescence of DCFH in all medication groups illustrated sharply elevated ·OH production, and higher ROS yield was exhibited in CMSFT+L or MCMSFT+L groups, owing to the photothermal effect accelerating the catalytic reaction rate and inducing more ROS generation (Fig. 5D and fig. S13A). Besides, the highest Cas-3 expression was induced by MCMSFT+L treatment, indicating that abundant ROS enhanced cellular apoptosis (Fig. 5B and fig. S11B). It is well known that GSH acts as a cofactor for GPX4 to catalyze lipid repair systems and GSH depletion can inactivate GPX4 to boost ferroptosis (*21*, *51*). Intracellular GSH and GPX4 as important antioxidant defenses to protect tumor cells from ferroptosis were measured. ThiolTracker violet staining indicated remarkable GSH depletion in all administration groups, and laser irradiation further exacerbated the reduction of intracellular GSH (Fig. 5E and fig. S13B). Western blotting results revealed the lowest level of GPX4 expression after MCMSFT+L treatment, hinting that increased GSH consumption and MET introduction were probably responsible for GPX4 expression inhibition (Fig. 5B and fig. S11C).

Apart from oxidative stress and redox homeostasis disruption, ferroptosis is also associated with iron dysmetabolism. Enhanced iron uptake and decreased iron storage can increase the labile iron pool (LIP), where ferrous ions are more potent inducers than ferric ions to boost effective ferroptosis (*51*, *52*). The intracellular ferrous level was measured by employing RhoNox-1 as a probe after various treatments. All medication groups observed distinct ferrous accumulation, meaning that the internalization of these formulations increased LIP to promote ferroptosis, through a noncanonical pathway independent of the typical mechanism of GPX4 inactivation (Fig. 5F and fig. S13C). Since ferritin is the major iron-storage protein within cells, the expression levels of ferritin were also examined after various treatments. The most serious ferritin degradation occurred in the MCMSFT+L group, implying that potentiated oxidative stress and photothermia contribute to the emancipation of reactive iron from ferritin to replenish the LIP, thereby strengthening ferroptosis (Fig. 5B and fig. S11D). Lethal accumulation of LPO is considered the gold-standard hallmark of ferroptosis. The fluorescent probe of C11-BODIPY was used to assess the intracellular LPO level after various treatments. As expected, the remarkable LPO accumulation displayed in the MCMSFT+L group implied the most violent ferroptosis (Fig. 5G and fig. S13D). These results supported that MCMSFT-mediated photothermally enhanced nanocatalysis and immunomodulation synergistic therapy operated by down-regulating PD-L1 expression, exacerbating oxidative stress, and intensifying GSH deprivation, thereby amplifying the hybrid multiple forms of cell death of apoptosis, ferroptosis, and necroptosis to optimize antitumor efficacy.

### ICD and immune stimulation

**Fig. 6.**
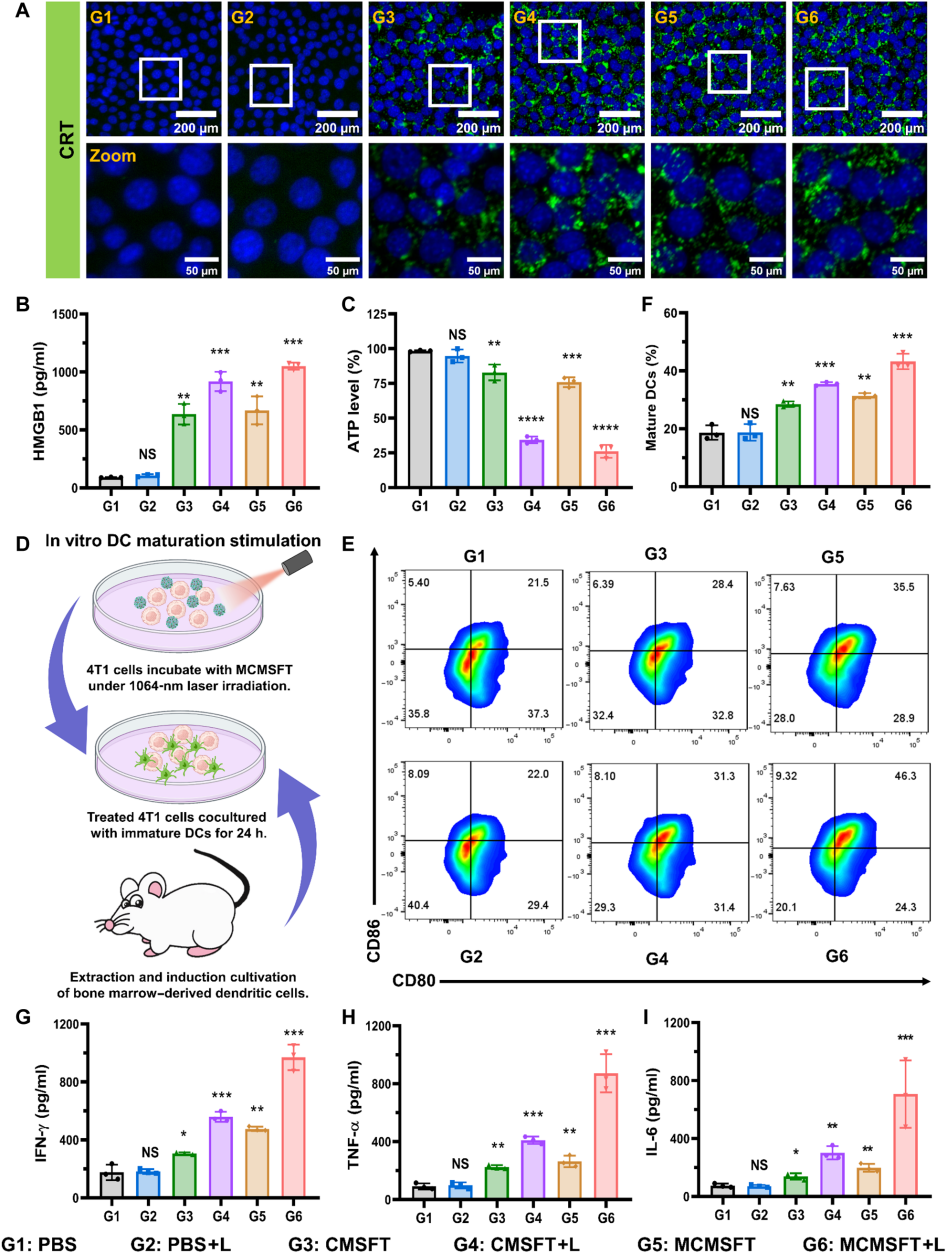
ICD and immune stimulation. (**A**) Surface-exposed CRT staining, (**B**) HMGB1 release measurement, and (**C**) Intracellular GSH level detection of 4T1 cells after different treatments. (**D**) Schematic illustration of various treated 4T1 cells in coculture with bone marrow DCs (BMDCs). (**E**) Representative flow cytometry plots and (**F**) quantitative analysis of mature DCs (CD11C^+^CD80^+^CD86^+^) after cocultivation. (**G** to **I**) Enzyme-linked immunosorbent assay (ELISA) measurement of IFN-γ, TNF-α, and IL-6 levels in cocultivation suspensions. The data are presented as the means ± SD, *n* = 3, *P < 0.05, **P < 0.01, ***P < 0.001, and ****P < 0.0001.

Extensive research uncovered that photothermal and ferroptosis treatment could induce tumor ICD, characterized by exposure of DAMPs, including calreticulin (CRT) surface translocation, the high-mobility group box 1 protein (HMGB1) secretion, and adenosine triphosphate (ATP) release, which promotes the antitumor immune response by stimulating DC maturation and T cell activation (*21*, *38*). Therefore, CRT surface exposure, HMGB1 secretion, and ATP release were detected in cells after various treatments to determine ICD induction. As shown in Fig. 6A, immunofluorescence images of CRT exhibited that cells treated with CMSFT or MCMSFT observed green fluorescence compared to the PBS group, and more prominent green fluorescence appeared after laser irradiation, indicating elevated cell surface exposure of CRT. The quantitative analysis of fluorescence intensity revealed that the highest CRT exposure occurred after MCMSFT+L treatment (fig. S14). Consistently, the highest level of HMGB1 secretion and the lowest level of intracellular ATP were witnessed in the MCMSFT+L group (Fig. 6, B and C). These results suggest that MCMSFT exhibited amplified ICD induction capability, attributed to the photothermal effect–aggravated ferroptosis via accelerated nanocatalytic reactions, which facilitated the awakening of the antitumor immune response.

The maturation of DCs is crucial in the initiation and maintenance of innate and adaptive immunity, and mature DCs secrete proinflammatory cytokines, IFN-γ, tumor necrosis factor–α (TNF-α), and interleukin-6 (IL-6), that stimulate T cell proliferation and differentiation to activate antitumor immunity (*21*, *36*, *38*). To evaluate the immune stimulation induced by MCMSFT, we established cocultivation systems to study the maturation of DCs after treatments in vitro (Fig. 6D). As shown in the representative flow cytometry plot and quantitative analysis (Fig. 6, E and F), MCMSFT+L treatment significantly promoted DC maturation, and the percentage of matured DCs was ~45%, more than doubled over the PBS group. Meanwhile, cytokines in cocultivation suspensions after different treatments were measured. As demonstrated in Fig. 6 (G and I), the highest cytokine secretion levels of IFN-γ, TNF-α, and IL-6 in the MCMSFT+L group were detected. These data indicated that MCMSFT-mediated photothermally enhanced nanocatalysis and immunomodulation synergistic therapy induced fierce ICD, efficiently promoted DC maturation, and potentially triggered robust antitumor immune responses.

### In vivo live imaging and therapeutic efficacy

**Fig. 7.**
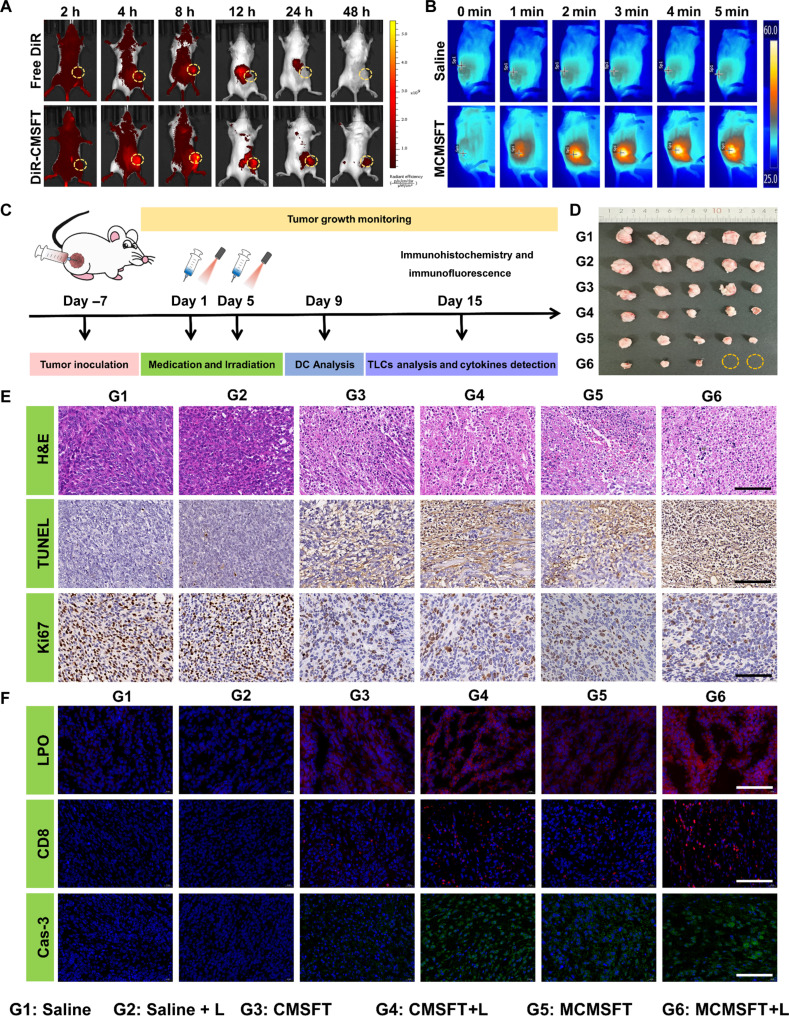
In vivo live imaging and therapeutic efficacy. (**A**) Fluorescence images of 4T1 tumor–bearing mice at different time points after intravenous administration of free DiR or DiR-CMSFT. (**B**) Time-dependent photothermal images of 4T1 tumor–bearing mice after intravenous administration of saline or MCMSFT with laser irradiation. (**C**) Schematic illustration of 4T1 subcutaneous tumor–bearing mouse model establishment and treatment schedule. (**D**) Photograph of tumors from mice after various treatments. (**E**) Representative hematoxylin and eosin (H&E), terminal deoxynucleotidyl transferase–mediated deoxyuridine triphosphate nick end labeling (TUNEL), and Ki67 staining images of tumor sections. Scale bars, 100 μm. (**F**) Representative LPO, CD8, and Cas-3 staining images of tumor sections. Scale bars, 100 μm.

Motivated by the marked therapeutic efficacy and remarkable immune stimulation effect of MCMSFT in vitro, further investigation of the in vivo antitumor therapeutic potential was put on the agenda. As shown in fig. S15, hemocompatibility assessment revealed that MCMSFT did not cause hemolysis even at higher concentrations, and the hemolysis rates of all groups were significantly lower than 5%, implying the favorable suitability for the intravenous administration of MCMSFT. Tumor diagnosis and treatment considerably benefited from adjuvant therapeutic information offered by nanomedicine bioimaging (*21*, *51*). Afterward, the biodistribution of MCMSFT was monitored in 4T1 subcutaneous tumor-bearing mice, free near-infrared lipophilic dye 1,1′-dioctadecyl-3,3,3′,3′-tetramethylindotricarbocyanine iodide (DiR) was set as a control group to simulate free drugs, fluorescently labeled DiR-CMSFT for real-time tracking of the in vivo biodistribution of nanoformulations. As visualized in Fig. 7A, the fluorescence signal at the tumor site of DiR-CMSFT was more intensive than that of free DiR, which was attributed to the typical enhanced permeability and retention effects. Moreover, preferential accumulation and prolonged retention at tumor sites were observed in the DiR-CMSFT group, compared to rapid clearance of the free DiR group. The quantitative fluorescence analysis (fig. S16A) proved that fluorescence signals showed a trend of gradual accumulation followed by continuous decline, with a peak at 8 hours postinjection in the DiR-CMSFT group, suggesting the optimal treatment window for laser irradiation. Furthermore, the ex vivo imaging and the corresponding quantification data (fig. S16, B and C) revealed the highest fluorescence intensity in tumors, further confirming excellent passive tumor-selective accumulation of DiR-CMSFT. Relative obvious fluorescence signals in the liver, kidney, and spleen may be due to metabolism and reticuloendothelial system absorption of nanoformulations. Subsequently, living photothermal imaging was performed in tumor-bearing mice 8 hours after MCMSFT injection. As presented in Fig. 7B and fig. S16D, negligible temperature variation was observed in mice in the saline group during laser irradiation. In contrast, mice in the MCMSFT group exhibited a prominent temperature elevation at the tumor site, which heated rapidly and maintained at ~50°C. These results suggest that MCMSFT can effectively accumulate at tumor sites and display outstanding photothermal properties in vivo, which makes vital sense for achieving a highly efficient antitumor effect.

The antitumor performance in vivo was then evaluated with 4T1 subcutaneous tumor–bearing mice as models (Fig. 7C). Tumor-bearing mice were randomly divided into six groups for different treatments: (i) saline, (ii) saline+L, (iii) CMSFT, (iv) CMSFT+L, (v) MCMSFT, and (vi) MCMSFT+L. Mice were intravenously administered an equal dosage of MCMSFT (10 mg/kg) on days 1 and 5. Then, the groups with laser treatment were exposed to the 1064-nm laser for 5 min at 8 hours postinjection. The tumor volumes and body weights of the mice were recorded every 3 days. As revealed by average and individual tumor growth curves in fig. S17 (A and B), compared to saline treatment groups, certain inhibition of tumor growth was observed in the CMSFT group owing to the limited NCT effect. Superior tumor growth arrest appeared in the group of CMSFT+L and MCMSFT, which was attributed to PTT-mediated thermal ablation and NCT enhancement or MET administration, resulting in more severe apoptosis/ferroptosis/necroptosis of tumor cells. Noticeably, the MCMSFT+L group displayed overwhelming tumor inhibition due to the multifunctional integration and even induced tumor regression. The photos and weights of tumors after different treatments are shown in Fig. 7D and fig. S17C. The lowest tumor weight and some tumor disappearance in the MCMSFT+L group illustrate that the treatment efficacy was much better than other groups. To further determine the in vivo therapeutic efficacy after various treatments, we used tumor sections for immunohistochemistry and immunofluorescence analysis (Fig. 7, E and F). Representative staining images of hematoxylin and eosin (H&E), terminal deoxynucleotidyl transferase–mediated deoxyuridine triphosphate nick end labeling (TUNEL), and Ki67 demonstrated the most profound cell apoptosis and necrosis and the lowest cell proliferation observed in tumor sections after MCMSFT+L treatment. Analogously, representative staining images of LPO, CD8, and Cas-3 confirmed that MCMSFT+L treatment induced excess LPO accumulation, increased immune cell infiltration, and enhanced apoptosis. These findings implicated that MCMSFT-mediated photothermally enhanced nanocatalysis and immunomodulation synergistic therapy exhibited remarkable antitumor performance, which exerted powerful antitumor effects through multiple pathways involving ferroptosis induction, apoptosis promotion, and immune activation.

The biosafety of MCMSFT was comprehensively estimated. As shown in fig. S18A, acceptable weight fluctuation hinted that medical interventions of all groups did not cause acute toxicity. Moreover, further histological H&E staining uncovered evident pathological change and tissue damage, which were hardly regarded in major organ sections after Saline or MCMSFT+L treatments (fig. S18B). Besides, the biochemical indices of liver and kidney function were within the normal range after MCMSFT+L treatment, whereas aberrantly elevated levels of aspartate aminotransferase and uric acid in the Saline group likely resulted from tumor burden, which caused abnormal metabolism (fig. S18C). These data proved that MCMSFT had excellent biosafety and held prospective promise for potential clinical translation.

### Ferroptosis-immune reciprocal effect and immune antitumor activity

**Fig. 8.**
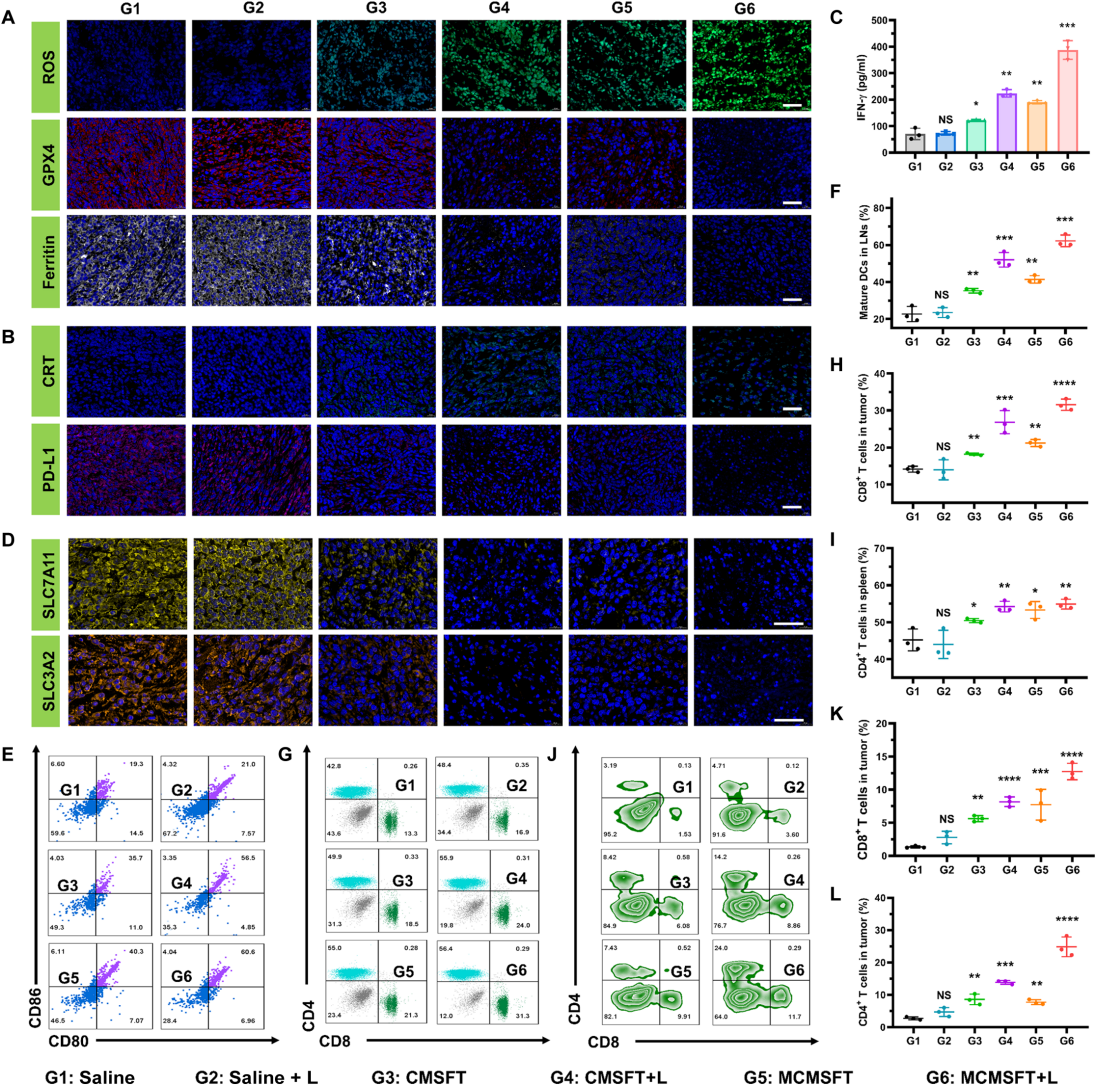
Ferroptosis-immune reciprocal effect and immune antitumor activity. (**A**) Representative fluorescence and immunofluorescence staining images of ROS, GPX4, and ferritin. Scale bars, 100 μm. (**B**) Representative immunofluorescence staining images of CRT and PD-L1. Scale bars, 100 μm. (**C**) ELISA measurement of IFN-γ levels in serum collected from mice after various treatments. (**D**) Representative immunofluorescence staining images of SLC7A11 and SLC3A2. Scale bars, 100 μm. (**E**) Representative flow cytometry plots and (**F**) quantitative analysis of mature DCs (CD11C^+^CD80^+^CD86^+^) in tumor-draining lymph nodes (LNs). (**G** to **L**) Representative flow cytometry plots and quantitative analysis of cytotoxic T lymphocytes (CD3^+^CD8^+^) and helper T lymphocytes (CD3^+^CD4^+^) in the spleen and tumor. The data are presented as the means ± SD, *n* = 3, *P < 0.05, **P < 0.01, ***P < 0.001, and ****P < 0.0001.

The updated research revealed a mutually reinforced interaction between ferroptosis and immunotherapy (*36*). Specifically, ferroptosis induces DAMPs and TAAs to activate antitumor immunity. Increased IFN-γ release from CD8^+^ T cells down-regulates SLC7A11 and SLC3A2 to arrest GSH synthesis, thereby disrupting GPX4-mediated LPO elimination and aggravating ferroptosis (*21*, *34*, *35*). The in vivo antitumor studies validated impressive ferroptosis and immune activation induced by MCMSFT-mediated synergistic therapy, encouraging further in-depth investigation to elucidate detailed regulatory mechanisms of the ferroptosis-immune reciprocal effect. On the basis of in vitro mechanisms studies, molecules associated with MCMSFT-mediated ferroptosis, including ROS, GPX4, and ferritin, were examined in tumor sections by fluorescence or immunofluorescence staining (Fig. 8A). Substantial ROS production, prominent GPX4 down-regulation, and forceful ferritin degradation in the MCMSFT+L group indicated enhanced LPO, reduced antioxidant defense, and increased LIP, respectively. This three-pronged mechanism resulted in overwhelming LPO accumulation to induce the most violent ferroptosis. Subsequently, immunofluorescence analysis of CRT and PD-L1 was performed to evaluate the immunostimulatory effect and the inhibition of immune evasion. As shown in Fig. 8B, the highest level of CRT exposure was determined in the MCMSFT+L group, meaning that the most intense ferroptosis could maximally reinforce immune activation. In addition, obvious PD-L1 down-regulation exhibited in the immunofluorescence image suggests that MCMSFT-mediated synergistic therapy significantly suppresses PD-L1–mediated immune evasion, rendering tumor cells more susceptible to immune cell attack. These results confirm that the MCMSFT-mediated synergistic therapy induced ferroptosis and enhances immunogenic stimulations conducive to promoting immune activation.

Immune activation is commonly associated with increased proinflammatory cytokines (*21*, *53*). The serum levels of proinflammatory cytokines of IFN-γ, TNF-α, and IL-6 were measured to assess the immune activation in vivo after various treatments (Fig. 8C and fig. S19). The results of enzyme-linked immunosorbent assays (ELISAs) illustrated that proinflammatory cytokines with varying degrees of increase in all medication groups. Moreover, the MCMSFT+L group showed the highest levels of IFN-γ, TNF-α, and IL-6, which implies that MCMSFT-mediated synergistic therapy may trigger strong immune responses. Recent research has demonstrated that the secretion of IFN-γ leads to the down-regulation of the cystine/glutamate antiporter system xc^−^ (SLC7A11/SLC3A2), which hinders the de novo synthesis of GSH to inactivate GPX4 triggers ferroptosis (*21*, *36*, *54*, *55*). To investigate the impact of immune activation on ferroptosis, we conducted the immunofluorescence analysis of SLC7A11 and SLC3A2. As illustrated in Fig. 8D, immunofluorescence-stained tumor sections confirmed the suppression of SLC7A11 and SLC3A2 expression in all medication groups. Moreover, the most pronounced reduction was observed in the MCMSFT+L group, underscoring the beneficial role of IFN-γ in promoting ferroptosis. Particularly noteworthy were loose tissues, enlarged intercellular gaps, reduced tumor cells, and nuclear fragmentation or disintegration in tumor sections, reflecting massive and vigorous tumor cell death. These results validated that MCMSFT-mediated synergistic therapy elicited expanded antitumor immunity and posed a positive feedback effect on ferroptosis, implying that ferroptosis-immune mutual amplification loop establishment is accountable for the superior in vivo antitumor performance.

The essence of tumor immunotherapy is to enhance antitumor immunity by targeting the cancer immunity cycle, which is a complex process orchestrated by multiple immune cells (*56*, *57*). To evaluate the immune antitumor activity, we investigated the phenotype and proportion of immune cells from mice after different treatments by flow cytometry analysis. The maturation of DC is crucial for promoting antigen presentation and priming T cell activation (*58*, *59*). As exhibited in Fig. 8 (E and F), the population of mature DCs in inguinal lymph nodes was prominently elevated to about 60% in the MCMSFT+L groups, validating that the MCMSFT+L treatment effectively promoted DC maturation. T cell–mediated tumor killing is predominant in antitumor immune response. The activation and tumor infiltration of T cells are highly relevant to immune antitumor activity (*21*, *38*). Flow cytometry analysis of T cells in the spleens was demonstrated in Fig. 8 (G to I), the population of cytotoxic T lymphocytes and helper T lymphocytes significantly increased after MCMSFT+L treatment in comparison with the saline group, suggesting that the MCMSFT-mediated synergistic therapy induces high levels of T cell activation. Furthermore, a similar tendency was observed in flow cytometry analysis of T cells in the tumors (Fig. 8, J to L), indicating abundant tumor infiltration of activated T cells. These data proved that the MCMSFT-mediated synergistic therapy evokes T cell–mediated forceful antitumor immune response and photothermally enhanced ferroptosis-immune reciprocal effect further boosts immune antitumor activity and improves the likelihood of tumor cure.

### Suppression of lung metastasis and rechallenge tumor

**Fig. 9.**
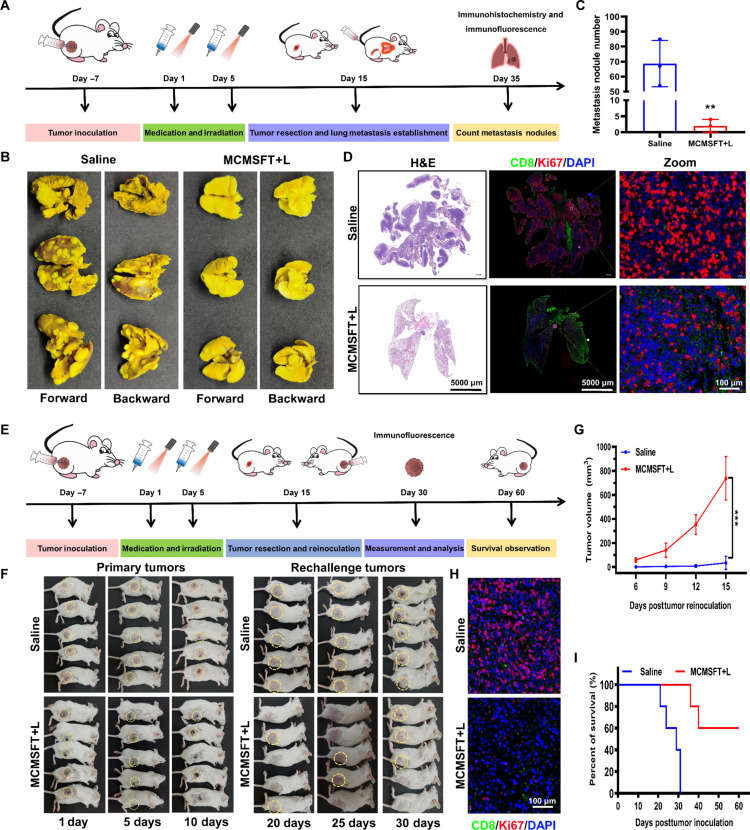
Suppression of lung metastasis and rechallenge tumor. (**A**) Schematic illustration of lung metastasis tumor–bearing mouse model establishment and treatment schedule. (**B** and **C**) Photographs of Bouin’s solution–stained lung tissues and quantitative analysis of metastasis nodules with different treatments. (**D**) H&E and immunofluorescence staining images of CD8 and Ki67 in the lung sections with different treatments. (**E**) Schematic illustration of rechallenge tumor–bearing mouse model establishment and treatment schedule. (**F**) Observation of primary tumors and rechallenge tumors growth with different treatments. (**G**) Quantitative analysis of the rechallenge tumor growth of mice with different treatments. (**H**) Immunofluorescence staining images of CD8 and Ki67 in the tumor sections with different treatments. (**I**) Survival curve of mice with different treatments. The data are presented as the means ± SD, *n* = 5, **P < 0.01 and ***P < 0.001.

In cancer treatments involving immunotherapy, the awakened immune system activates powerful immune responses to destroy primary tumors and induce systemic and long-term antitumor immunity to inhibit tumor metastasis and relapse (*21*, *35*, *38*, *60*). Tumor metastasis terribly increases the morbidity and mortality of cancer, and highly invasive breast cancer is prone to lung metastasis. The lung metastasis tumor model was constructed as described in Fig. 9A to assess MCMSFT-induced systemic antitumor immunity by examining the inhibition effect of lung metastasis formation. Photographs of Bouin’s solution–stained lung tissues and quantitative analysis of metastasis nodules were demonstrated in Fig. 9 (B and C), and almost no metastatic nodules were observed in the MCMSFT+L group compared to countless nodules in the saline group. Moreover, as H&E and immunofluorescence staining images were shown in Fig. 9D, extensive tumor lesion occupation and horrible tumor cell proliferation occurred in the saline group. Whereas tumor metastasis was effectually controlled in the MCMSFT+L group, restricted cell proliferation and increased immune cell infiltration were observed even in rare lesion sites. These results indicated that MCMSFT+L treatment effectively reduced lung metastasis formation, attributed to MCMSFT-induced systemic fierce antitumor immunity hunting and eradicating the metastatic tumor cells.

Tumor recurrence recognized as another bottleneck in clinical cancer treatment contributes to the poor prognosis and low survival rate. The rechallenge tumor model was established as illustrated in Fig. 9E to evaluate MCMSFT-induced long-term antitumor immunity by monitoring suppression of rechallenge tumor growth. As depicted in Fig. 9F, some of the primary tumors in the MCMSFT+L group progressively disappeared after the cessation of medical intervention, meaning that immune activation facilitated the killing of the residual tumor cells. The digital photographs and quantitative analysis of rechallenge tumor growth (Fig. 9, F and G) validated the rapid formation of tumor foci in all mice in the saline group and the explosive increase in tumor volume. In contrast, rechallenge tumor growth was profoundly inhibited in the MCMSFT+L group, and some mice never evolved a rechallenged tumor by the end of the observation period. Immunofluorescence staining images of the rechallenged tumor section indicated vigorous tumor cell proliferation in the saline group, while the tumor section from the CMSFT+L-treated mice showed increased immune cell infiltration (Fig. 9H). The survival status of the mice was recorded for survival curve creation, which disclosed notable differences in survival between the saline and MCMSFT+L groups (Fig. 9I). Around 30 days after treatments, most of the mice in the saline group died spontaneously from tumor burden or had to be euthanized because of tumor overload and fragile states. In contrast, some mice in the MCMSFT+L group survived for more than 60 days, with a survival rate of up to 60% by the end of the observation period. Furthermore, spleens were harvested from mice on day 30 for flow cytometry analysis to determine specific immune cell populations involved in long-term antitumor immunity (fig. S20). Representative flow cytometry plots and quantitative analysis showed that the proportions of central memory T cells and effector memory T cells were significantly increased in the MCMSFT+L group, denoting that MCMSFT potentially motivated tumor-specific immune memory and built a persistent defense to safeguard mice from tumor reattack. These findings proved that MCMSFT-mediated photothermally enhanced nanocatalysis and immunomodulatory synergistic therapy fully activated the immune antitumor effect, established a systemic and long-term immune response, and threatened the lung metastasis formation and the rechallenge tumor growth, implying the exceptional promise of MCMSFT in inhibiting tumor metastasis and recurrence.

## DISCUSSION

In summary, the MCMSFT with CAT/POD/GPx-mimicking properties was constructed in this work. MCMSFT exhibited photothermally enhanced multienzyme activities for initiating hybrid cell death of apoptosis/ferroptosis/necroptosis. By intelligently using the unique features of TME and the cycling valence alternation of metal ions, MCMSFT achieved highly efficient tumor NCT. MCMSFT provided sustained oxygen generation, and MET release favored PD-L1 down-regulation to relieve immune evasion. Meanwhile, continuous ·OH production and GSH consumption induced by MCMSFT disrupt intracellular redox homeostasis and inactivate the LPO-scavenging function of GPX4, aggravating apoptosis and ferroptosis. Moreover, the outstanding photothermal property of MCMSFT enabled effective tumor thermal ablation, and PTT-associated temperature increase accelerated nanocatalytic reactions to amplify apoptosis and ferroptosis. In addition, PTT and ferroptosis caused ICD, stimulating DC maturation and T cell activation to reinforce antitumor immunity. Subsequent IFN-γ secretion arrested the expression of SLC3A2 and SLC7A11 and further boosted ferroptosis. In vitro and in vivo studies illustrated that multifunctional nanozyme MCMSFT displayed satisfactory performance in eliminating primary tumors and suppressing metastasis/rechallenge tumors. Thus, MCMSFT-mediated hyperthermally promoted nanocatalysis and immunomodulation reciprocal synergistic therapy have prominent antitumor efficacy by overcoming apoptosis resistance and immune evasion in cancer treatment.

## MATERIALS AND METHODS

### Materials and reagents

Copper(II) chloride dihydrate (CuCl_2_·2H_2_O), sodium hydroxide (NaOH), ascorbic acid, glucose, sodium molybdate dihydrate (Na_2_MoO_4_·2H_2_O), TA, iron(III) chloride hexahydrate (FeCl_3_·6H_2_O), and thioacetamide (C_2_H_5_NS) were purchased from Aladdin (Shanghai, China). Polyvinylpyrrolidone (PVP) and DFO were purchased from Sigma-Aldrich (St. Louis, MO, USA). RhB, ethanol, NaOH, hydrochloric acid (HCl), and ethylene glycol were purchased from Chengdu Kelong Chemicals (Chengdu, China). MET, Fer-1, and DEVD were purchased from MedChemExpress (Shanghai, China). Cell Counting Kit-8 assay kit, calcein/propidium iodide (PI) staining assay kit, nuclear dye 4′,6-diamidino-2-phenylindole (DAPI), Hoechst 33342 (Hoechst), 3,3′,5,5′-TMB, DTNB, DCFH-diacetate (DCFH-DA), LysoTracker green, JC-1 MMP assay kit, and ATP assay kit were purchased from Beyotime Biotechnology (Shanghai, China). C11-BODIPY was purchased from Thermo Fisher Scientific (Waltham, MA, USA). ThiolTracker violet was purchased from ATT Bioquest (Sunnyvale, CA, USA). [Ru(dpp)_3_]Cl_2_ and FeRhoNox-1 were purchased from MaokangBio (Shanghai, China). Near-infrared fluorescence dye DiR was purchased from Abbkine Scientific (Wuhan, China). ELISA kit was purchased from NeoBioscience (Shenzhen, China). Antibodies for Western blot and immunofluorescence were bought from Cell Signaling Technology (Danvers, MA, USA). Antibodies for flow cytometry analysis were acquired from BD Pharmingen (San Diego, CA, USA). All chemicals and reagents were used as received according to the manufacturer’s instructions.

### Characterization and apparatus

TEM images and STEM-EDX element mapping images were acquired on a field emission electron microscope (Tecnai G2 F20 S-Twin, FEI, USA). Zeta potential and dynamic light scattering were measured by Zetasizer nanoseries (Nano ZS90, Malvern, UK). Pore size distribution and nitrogen adsorption-desorption isotherm were determined by Brunauer-Emmett-Teller surface area and porosity analyzer (TriStar II 3020, Micromeritics, USA). Elemental compositions and valence states were probed by XPS (K-Alpha, Thermo Fisher Scientific, USA). XRD pattern was recorded by diffractometer (XRD-6100, Shimadzu, Japan). The infrared spectra were obtained by FTIR spectroscopy (Nicolet iS5, Thermo Fisher Scientific, USA). The absorbance was monitored by ultraviolet-visible–near-infrared spectrophotometer (Lambda 750, PerkinElmer, USA) or Microplate Reader (Multiskan Sky, Thermo Fisher Scientific, USA). The dissolved oxygen concentration was reported by a dissolved oxygen meter (ray magnetic, JPSI-605F, China). NIR-II irradiation was provided by a 1064-nm laser (WPL1-Y1009-A22, wave-particle laser, China). The temperature variation was probed by thermocouples (HH806AU, Omega Engineering, USA). Photothermal images were captured by the infrared thermal camera (E54 Series, FLIR, USA).

### Synthesis of MCMSFT

To obtain CMS, 0.191 g of CuCl_2_·2H_2_O and 2.54 g of PVP were dispersed in 80 ml of ultrapure water. Subsequently, NaOH aqueous solution (2 M, 10 ml) and ascorbic acid aqueous solution (0.6 M, 10 ml) were serially added dropwise. After rigorous stirring for 1 hour, the products were collected by centrifugation (10,000 rpm for 5 min) and washed with ultrapure water and ethanol several times to acquire Cu_2_O. Afterward, 10 mg of Cu_2_O and 30 mg of glucose were dispersed in 20 ml of ethylene glycol and sonicated for 30 min. Then, 17.5 mg of Na_2_MoO_4_·2H_2_O and 30 mg of C_2_H_5_NS were added into the solution and sonicated for another 30 min. The mixed solution was transferred into a Teflon-lined stainless-steel autoclave and maintained at 160°C for 12 hours. Products were collected by centrifugation (10,000 rpm for 5 min) and washed several times with ultrapure water and ethanol to obtain CMS.

To fabricate MCMSFT, 10 mg of CMS and 10 mg of MET were dispersed in 10 ml of ultrapure water and stirred for 48 hours. The products were collected by centrifugation (10,000 rpm for 5 min) and washed with ultrapure water. The above products were resuspended with 10 ml of ultrapure water, followed by dropwise addition of TA solution (40 mg/ml, 250 μl) and FeCl_3_ solution (10 mg/ml, 125 μl) under sonication. Subsequently, neutralization to form metal polyphenol networks with NaOH solution (0.1 M). The products were collected by centrifugation (8000 rpm for 15 min) and washed several times with ultrapure water and ethanol to gain MCMSFT. The preparation of empty CMSFT or fluorescence-labeled RhB-CMSFT and DiR-CMSFT followed the same procedure except without MET loading or using fluorescent dye RhB and DiR to replace MET.

### Cell lines and culture

Murine breast cancer cell line 4T1 and EMT-6 cells and mouse embryonic fibroblast cell line NIH3T3 cells were purchased from the Cell Bank of the Chinese Academy of Sciences (Shanghai, China). Murine bone marrow DCs (BMDCs) were acquired as previous works described (*21*, *61*). RPMI 1640 and Dulbecco’s modified Eagle’s medium (DMEM), fetal bovine serum (FBS), penicillin-streptomycin (PS), and other cell culture–associated products purchased from Gibco (Billings, MT, USA). 4T1 and EMT-6 cells were cultured with RPMI 1640, and NIH3T3 cells were cultured with DMEM, both containing 10% FBS and 1% PS. All cells were cultured in a humidified atmosphere with 5% CO_2_ at 37°C.

### Tumor model establishment

Female Balb/c mice aged 5 weeks were purchased from the Experimental Animal Center of Sichuan University (Chengdu, China). The primary subcutaneous tumor model was established by injecting 4T1 cells (2 × 10^6^ cells suspended in 100 μl of saline) subcutaneously into the right hind-limb region of the mice. When tumors reached a volume of ~100 mm^3^ (volume = length × width^2^ / 2), mice were randomly assigned to several groups for further use (*n* = 5). The saline- and MCMSFT+L–treated subcutaneous tumor–bearing mice thoroughly removed the subcutaneous residual tumor on day 15. Postoperative mice injected 4T1 cells (1 × 10^6^ cells) intravenously to develop the lung metastasis model. Similarly, postoperative mice reinjected 4T1 cells (2 × 10^6^ cells) subcutaneously in the contralateral site of the primary tumor to develop the rechallenge tumor model. All animal research was performed in agreement with the guidelines of the Institutional Animal Care and Ethics Committee of the University of Electronic Science and Technology of China (approval number: 1061423022725123). During all in vivo imaging and surgical procedures, mice were anesthetized with isoflurane. More experimental methods are available in the Supplementary Materials.
