# Supplementary material for: Endogenous/exogenous dual-responsive nanozyme for photothermally enhanced ferroptosis-immune reciprocal synergistic tumor therapy
Source: Sci Adv. 2025 May 14;11(20):eadq3870. doi: 10.1126/sciadv.adq3870 (PMC12077522; doi:10.1126/sciadv.adq3870)
Supplement: Supplementary file 1 — Supplementary Text Figs. S1 to S20 [file sciadv.adq3870_sm.pdf]

Science Advances
AAAS

Supplementary Materials for

Endogenous/exogenous dual-responsive nanozyme for photothermally enhanced ferroptosis-immune reciprocal synergistic tumor therapy

Hanxi Zhang *et al.*

Corresponding author: Dong Lv, lv-0919@163.com; Yiyao Liu, liuyiyao@uestc.edu.cn;
Hong Yang, yanghongyh@uestc.edu.cn

Sci. Adv. **11**, eadq3870 (2025)
DOI: 10.1126/sciadv.adq3870

This PDF file includes:

Supplementary Text
Figs. S1 to S20

Experiment Section

Drug loading and release

In the process of MCMSFT synthesis, the drug loading efficiency (DL%) and drug encapsulation efficiency (EE%) of MET were detected by high-performance liquid chromatography (HPLC). The DL% and EE% were calculated using the following formulas: DL% = (mass of the drug in formulation/mass of the formulation) × 100%, EE% = (mass of the drug in formulation/mass of the feeding drug) × 100%. To explore the MET release behavior, the dialysis bag diffusion method was used to assess the *in vitro* drug release profile of the MCMSFT. Specifically, MCMSFT (10 mg, 1mL) was encapsulated into a dialysis bag (molecular weight of 5000), which was sealed and immersed in the PBS buffer with different pH (5.0, 6.5, and 7.4) at a volume of 9 mL. The above drug release system was placed in a constant temperature water bath at 37 °C to maintain continuous magnetic stirring. According to the set time points (2, 4, 6, 8, 16, 24, 36, 48, 60, and 72 h), 1 mL of the solution outside the dialysis bag was drawn out respectively. Finally, the withdrawn solution was detected by HPLC, and the mass of MET released by MCMSFT at each time point was calculated. The mass of MET released from MCMSFT at different time points was summed up to calculate the cumulative release of MET at each time point. The drug release with laser irradiation was performed following the same process except a laser irradiation pretreatment for 5 min.

Biodegradation behavior

To evaluate the possible disintegration characteristics of MCMSFT, the biodegradation behavior of MCMSFT was studied in PBS buffers mimicking physiological conditions (pH 7.4) and tumor milieu conditions (pH 6.5 in tumor microenvironments or pH 5.0 in lysosomes with 10 mM GSH and 100 μM H2O2). MCMSFT (100 μg/mL) was incubated with the above buffers at 37 °C for 72 h. Afterward, the precipitate was collected from each group for the morphology observation using TEM.

Extracellular photothermal performance examination

To investigate the photothermal capability, different concentrations of MCMSFT solution (0, 25, 50, 100, and 200 μg/mL, 200 μL) exposed to 1064 nm laser with various power densities (0, 0.25, 0.5, and 1 W/cm^2) for 5 min. The thermocouple was used to measure the real-time temperature, and the temperature variation was recorded every 30 s. The photothermal conversion efficiency (η) of MCMSFT was calculated using cooling stages data, referring to reported equations in previous literature. Likewise, different concentrations of MCMSFT solutions (0, 25, 50, 100, and 200 μg/mL,

1mL) were placed in centrifuge tubes and exposed to 1064 nm laser. The infrared thermal camera was used to capture photothermal images at preset time points. To assess the photothermal stability, MCMSFT solution (100 μg/mL, 200 μL) was exposed to 1064 nm laser (0.5 W/cm^2) for 5 min followed by natural cooling for another 5 min after removal of laser irradiation. The above procedure was repeated five times, and the temperature variation was recorded.

Extracellular multienzyme-like activity detection

The CAT-like enzyme activity of MCMSFT was detected by monitoring oxygen generation.MCMSFT solution (100 μg/mL, pH 5.0) was added with H_2O_2 (100 μM). Once H_2O_2 was added, the oxygen concentration began to be monitored by the dissolved oxygen meter. The POD-like enzyme activity of MCMSFT was detected by investigating •OH production. MCMSFT solution (100 μg/mL, pH 5.0) was added with H_2O_2 (100 μM) and TMB (1 mM) and incubated for 30 min. The absorbance of the supernatant solution was recorded using the UV-vis spectrophotometer. The GPx-like enzyme activity of MCMSFT was valued by examining GSH consumption. MCMSFT solution (100 μg/mL, pH 5.0) was added with GSH (10 mM) and incubated for 30 min, followed by mixing with DTNB (1 mM) for 10 min. The absorbance of the supernatant solution was recorded using the UV-vis spectrophotometer. The samples plus laser irradiation were performed in the same process.

Cell cytotoxicity evaluation

The cytotoxicity was evaluated using a cell counting kit (CCK-8). NIH 3T3 cells were seeded into 96-well plates and cultured overnight. Subsequently, the cells were incubated with different concentrations (0, 25, 50,100, and 200 μg/mL) of MCMSFT for 24 h. The cell viability was measured by CCK-8 assay according to the manufacturer’s instructions. The same procedures were simultaneously performed in EMT-6 and 4T1 cells.

Cellular uptake and lysosome colocalization

To investigate the cellular uptake of nanoformulations, 4T1 cells were seeded into 6-well plates cultured overnight and then incubated with RhB-CMSFT for different time intervals. The cells were washed with PBS several times and fixed with 4% paraformaldehyde. After that, the cells were stained with DAPI. The cellular uptake of nanoformulation was observed using fluorescence microscopy (Eclipse Ti2-E, Nikon, Japan). The quantitative analysis of cellular uptake was conducted by measuring the fluorescence intensity by flow cytometry (NovoCyte Quanteon, Agilent, USA). To visualize the intracellular distribution of nanoformulations, 4T1 cells were

incubated with RhB-CMSFT for 4 h and washed with PBS several times. Subsequently, the cells were stained with LysoTracker Green and Hoechst for 30 min before fluorescence microscopy observation.

Regimen of treatments

For the *in vitro* treatment experiment, cell samples were basically divided into the following six groups: (1) PBS, (2)PBS+L, (3) CMSFT, (4) CMSFT+L, (5) MCMSFT+L, and (6) MCMSFT+L. Medication at an equal dose of MCMSFT (100 μg/mL). 4T1 cells were seeded and cultured overnight, followed by incubation with the corresponding formulation for 12 h, after which the medium was replaced with fresh culture medium. Laser irradiation (1064 nm, 0.5 W/cm^2, 5 min) was immediately performed in the required groups. All cell samples were continued to be cultured for another 24 h. Cell samples finished the above protocol were used for further experimental observations and examination.

For the *in vivo* treatment experiment, tumor-bearing mice were essentially assigned to the following six groups: (1) Saline, (2)Saline+L, (3) CMSFT, (4) CMSFT+L, (5) MCMSFT+L, and (6) MCMSFT+L. Administration at an equal dose of MCMSFT (10 mg/kg). The respective formulation of each group was administered intravenously into tumor-bearing mice. Laser irradiation (1064 nm, 0.5 W/cm^2, 5 min) was performed in the required groups 8 h after post-injection. Medication twice in total with a 3-day interval.

Hybrid cell death induction

To examine the hybrid cell death induction, treated 4T1 cells performed caspase-3 (Cas-3) immunofluorescence and lipid peroxidation (LPO) staining to determine the coexistence of apoptosis and ferroptosis. 4T1 cells after PBS or MCMSFT treatments were washed with PBS and then fixed with 4% paraformaldehyde. Subsequently, cell samples were processed for the Cas-3 standard immunofluorescence staining protocol. Then, cell samples were sequentially stained with C11-BODIPY (10 μM) and DAPI (10 μM) for 30 min. Cell staining images were visualized by fluorescence microscopy. To further verify the induction of ferroptosis and apoptosis, the effect of ferroptosis and apoptosis inhibitors on cell viability was measured. 4T1 cells were incubated with MCMSFT (100 μg/mL) added 100 μM DFO, 10 μM Fer-1, and 50 μM DEVD, respectively. After 24 h of incubation, the CCK-8 assay was performed to determine cell viability.

In vitro antitumor effect assessment

The *in vitro* antitumor effect was assessed by investigating the cell viability of 4T1 cells finished various treatment procedures of (1) PBS, (2)PBS+L, (3) CMSFT, (4) CMSFT+L, (5) MCMSFT+L, and (6) MCMSFT+L. The CCK-8 assay and the live/dead cell staining were performed to analyze the cell viability according to the manufacturer's instructions.

Intracellular oxygen production

Oxygen sensor $[Ru(dpp)_3]Cl_2$ was employed to detect intracellular oxygen production. 4T1 cells were seeded into 6-well culture plates and cultured for 24 h under hypoxic conditions. Followed by cells incubated with $[Ru(dpp)_3]Cl_2$ (5 μM) for 4 h and treated with PBS or MCMSFT (100 μg/mL) for another 4 h. Finally, the fluorescent signal of $[Ru(dpp)_3]Cl_2$ in cells were visualized by fluorescence microscopy.

Mitochondrial morphology and membrane potential observation

The morphology and membrane potential of mitochondria were observed by biological electron microscopy (Bio-TEM) and JC-1 staining. 4T1 cells were seeded and cultured overnight, followed by treated with PBS or MCMSFT for 24 h. Treated cells were collected and fixed with glutaraldehyde for Bio-TEM observation. Simultaneously, the membrane potential of 4T1 cells finished various treatment procedures was stained with the JC-1 staining kit following the protocol of the manufacturer and visualized by fluorescence microscopy.

Western blotting analysis

4T1 cells finished various treatment procedures, cell samples were collected and lysed using RIPA lysis and extraction buffer with 1% proteinase and phosphatase inhibitors referring to the manufacturer’s instructions. Protein samples were collected from the supernatants and quantified by bicinchoninic acid (BCA) analysis. Subsequently, the equivalent protein was mixed with the protein loading buffer and boiled for 10 min. Equal amounts of each sample were applied to 10% SDS–PAGE and subjected to electrophoresis. Then, the proteins were transferred to polyvinylidene difluoride (PVDF) membranes and blocked with 5% skimmed milk buffer for 2 h at room temperature. Afterward, PVDF membranes were incubated overnight at 4 ◦C with primary antibodies (PD-L1, Cas-3, GPX4, Ferritin) and then secondary antibodies according to the manufacturer’s recommendations. The chemiluminescence imaging was acquired with the Chemiluminescent Imaging System (5200, Tanon, China). Western blot band quantifications were normalized to GAPDH housekeeping gene levels, and the intensity of the western blotting bands was quantified using ImageJ software (Fiji ImageJ, Maryland, USA).

Intracellular ROS, GSH, Fe^{2+} and LPO levels detection

After 4T1 cells finished various treatment procedures, for ROS assay, the cells were stained with DCFH-DA (10 μM) for 30 min, nuclear staining was performed with Hoechst (10 μM) simultaneously. Images were acquired using a fluorescence microscope. For GSH assay, treated cell samples were stained with ThiolTrace violet (5 μM) for 30 min and imaged with a fluorescence microscope. For Fe^{2+} and LPO assay, treated cell samples were fixed and stained with FeRhoNox-1 (5 μM) or C11-BODIPY (10 μM) for 30 min, nuclear staining was performed with DAPI (10 μM), fluorescence images captured by fluorescence microscope. Corresponding quantitative analysis was carried out by flow cytometry and GSH assay kit according to the manufacturer's protocol.

Immunogenic cell death measurement

4T1 cells after different treatments were cultured for another 24 h. Calreticulin (CRT) exposure was measured by immunofluorescence staining and flow cytometry. Cells were fixed and incubated with anti-CRT primary antibody at 4°C overnight, followed by Alexa Fluor 488 conjugated secondary antibody incubation for 2 h at room temperature. The cells were then observed by fluorescence microscopy and analyzed by flow cytometry. High-mobility group box 1 (HMGB1) protein releases and intracellular adenosine triphosphate (ATP) levels were assessed by the HMGB1 ELISA Kit and ATP Assay Kit, respectively. Cell supernatants and lysates were collected for detection according to the manufacturer's protocol.

Dendritic cell maturation stimulation

Bone marrow-derived DCs were acquired as previous works described. 4T1 cells were cultured for another 24 h after different treatments. DCs were collected and co-cultured with treated 4T1 cells mentioned above for an additional 24 h. Non-adherent cells were collected and stained with anti-CD11c-BB700, anti-CD80-APC, and anti-CD86-PE antibodies. Fluorescence-activated cell sorting (FACS) was performed to analyze the maturation of DCs. The supernatant of the cell's co-culture medium was collected and the cytokine levels of IFN-γ, TNF-α, and IL-6 were detected using an ELISA kit according to the manufacturer’s instructions.

Hemolysis assay

The hemolysis experiments were measured following standard procedures. The nanoformulation was mixed with mouse red blood cell suspension (2% in saline). The final

concentrations of MCMSFT in RBC suspensions were 25-300 μg/mL. Then, the mixtures were shaken at 37 °C for 2 h and then centrifuged at 3000 rpm for 10 min. To determine the hemoglobin concentration of the supernatant, a microplate reader was used to measure the absorbance at 545 nm. Water and saline were chosen as the positive and negative controls.

Biodistribution and in vivo photothermal imaging

In/ex vivo fluorescence imaging was performed to investigated the biodistribution of MCMSFT in the subcutaneous tumor-bearing mice. The mice were intravenously injected with free DiR or DiR-CMSFT at the dose of 2 mg/kg DiR (Ex/Em: 748/780 nm), respectively. Fluorescence imaging was conducted using an IVIS Lumina Series III imaging system (PerkinElmer, USA) at predetermined time points (2, 4, 8, 12, 24, and 48 h). Then, the major organs (heart, liver, spleen, lung, and kidney) and tumor tissues of the mice were harvested for *ex vivo* imaging.

In vivo photothermal performance of MCMSFT was evaluated by photothermal imaging in the subcutaneous tumor-bearing mice. The mice were intravenously injected with saline or MCMSFT, and the laser irradiation (1064 nm, 0.5 W/cm^2, 5 min) was imposed on the tumor site at 8 h after administration. The infrared thermal imaging camera was used to record the thermal images and local temperature variations.

In vivo therapeutic efficacy investigation

The 4T1 subcutaneous tumor-bearing mice accepted a series of treatments, tumor volume and body weight were monitored every 3 days. At the endpoint, mice were sacrificed and tumors were excised and photographed. Tumor tissues were embedded and sectioned for further immunohistochemistry and immunofluorescence assay. On day 9th, the inguinal lymph nodes were harvested for flow cytometry analysis to assess *in vivo* DC maturation. On day 15th, the spleen and tumor were accumulated for flow cytometry analysis to evaluate the activation of T cells, serum was also collected for cytokine detection. The phenotypic analysis of T lymphocytes by staining cells with anti-CD3-FITC, anti-CD4-PE, and anti-CD8-APC antibodies, and analyzed by FACS Canto II flow cytometry (BD Biosciences, USA).

Lung metastasis and rechallenge tumor suppression

The lung metastasis tumor-bearing mice were sacrificed on day 35th, lungs were harvested and kept in Bouin's fixative solution overnight to detect metastasis. The pulmonary metastatic nodules were counted and analyzed. Lung tissues were embedded and sectioned for further immunohistochemistry and immunofluorescence assay.

Half of the rechallenge tumor-bearing mice from each group were sacrificed on day 30th, spleen and tumor were harvested for flow cytometry analysis and immunofluorescence assay. The phenotypic analysis of memory T cells by staining cells with anti-CD3-FITC, anti-CD8-APC, anti-CD44-Cy5.5, and anti-CD62L-PE antibodies. Flow cytometry data were analyzed by FlowJo v10 software (FlowJo LLC, USA). The survival status of the other half mice was recorded until day 60th, mice were sacrificed when tumor volume exceeded 1000 mm^3, survival rate of the mice was monitored and calculated.

Biosafety evaluation

At the end of the animal experiments, blood samples drawn from saline and MCMSFT+L groups were performed biochemistry analysis. The hepatorenal function biochemical index including alkaline phosphatase (ALP), alanine aminotransferase (ALT), aspartate aminotransferase (AST), uric acid (UA), urea (UREA), and creatinine (CREA) was measured using the serum samples by blood biochemistry analyzer (Mindray, China).

Statistical analysis

Each experiment for statistical analysis was independently replicated at least three times. Data were presented as the mean ± standard deviation (SD) using GraphPad Prism software version 10 (GraphPad Software Inc., San Diego, CA, USA). The corresponding figure legend indicated the sample sizes (n) for statistical calculation. Group comparisons were performed using the two-tailed Student's *t*-test or one-way analysis of variance (ANOVA) with post hoc Tukey's test. $p < 0.05$ was considered statistically significant, with levels of significance denoted as $*p < 0.05$, $**p < 0.01$, $***p < 0.001$, and $****p < 0.0001$.

Supplementary Figures

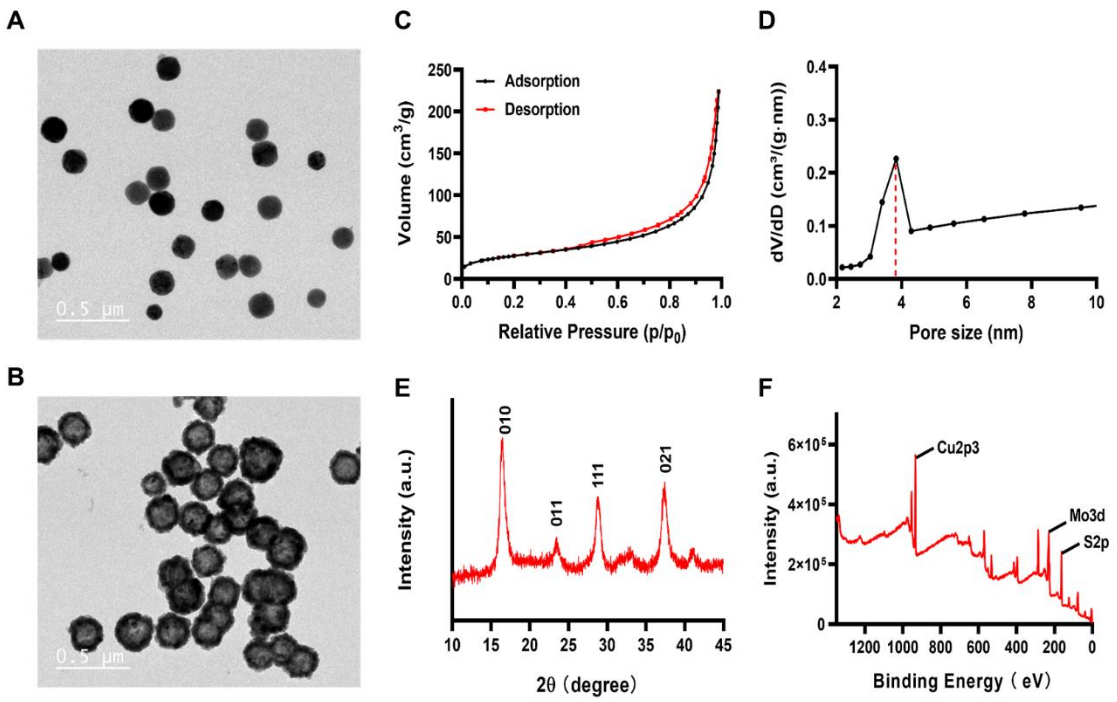

Fig. S1.

Characterization of CMS. (**A**) TEM image of Cu_2O. (**B**) TEM image of CMS. (**C**) The nitrogen adsorption and desorption isotherms of CMS. (**D**) The corresponding pore size distribution of CMS. (**E**) The XRD pattern of CMS. (**F**) The XPS spectra of CMS.

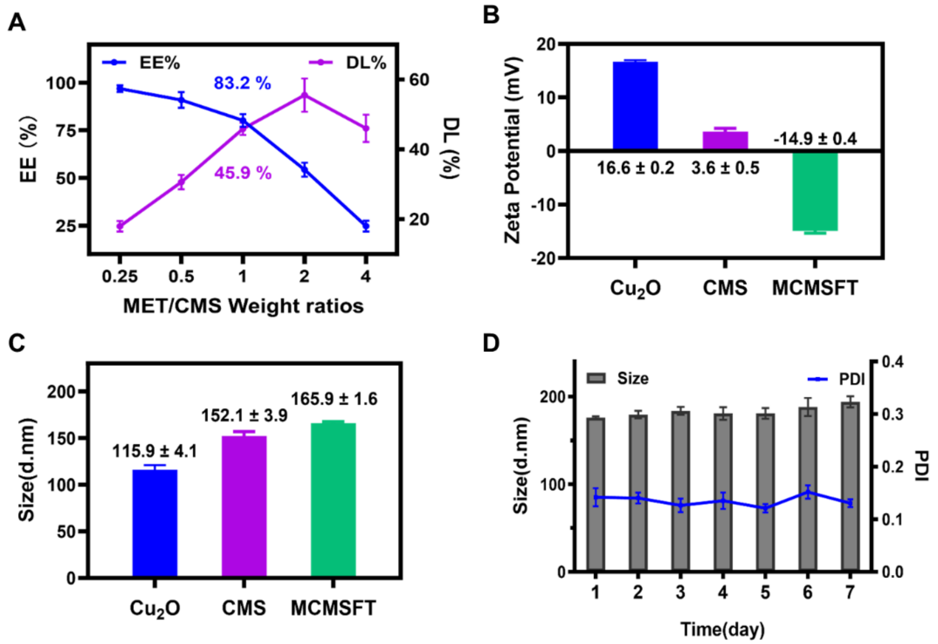

Fig. S2.

Characterization of MCMSFT. (**A**) Encapsulation efficiency and drug loading of MET at various drug feeding rates. (**B**) Hydrodynamic particle size and (**C**) Zeta potential measurement of Cu_2O, CMS, and MCMSFT. (**D**) The variation in particle size and PDI of MCMSFT with time duration in PBS buffer. The data are presented as the mean ± SD, n = 3.

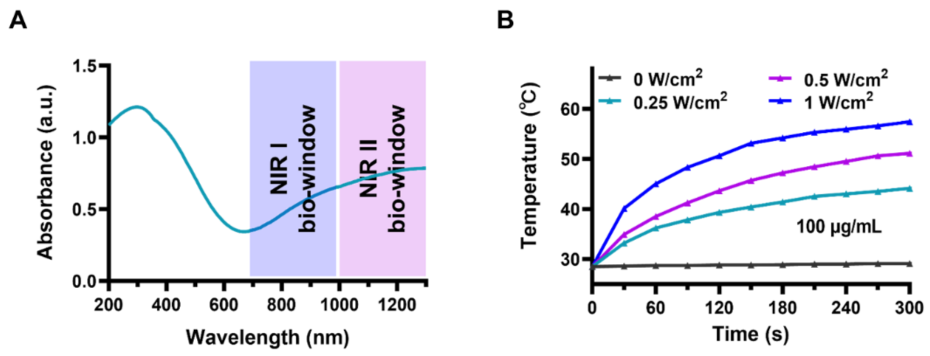

Fig. S3.

Photothermal property of MCMSFT. (**A**) Vis/NIR absorption spectra of MCMSFT. (**B**) Photothermal heating curves of MCMSFT with laser irradiation at different power densities for 5 min.

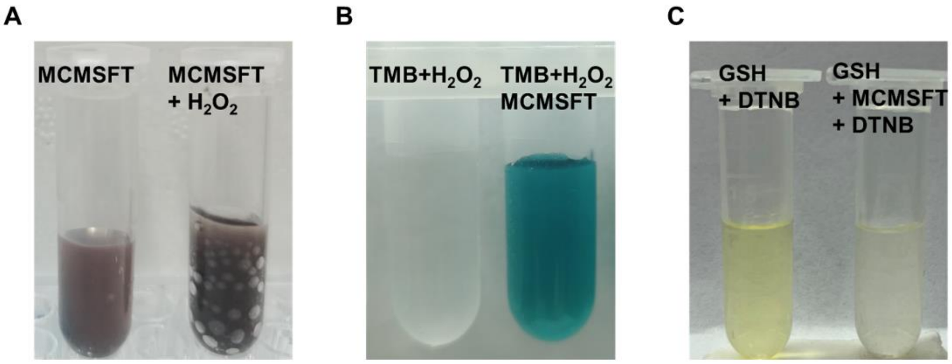

Fig. S4.

Multienzyme-like activity of MCMSFT. (**A**) The phenomenon of oxygen generation for MCMSFT after 2 h of reaction without or with H_2O_2. (**B**) The observation of color change in the TMB assay after 2 h of reaction without or with MCMSFT. (**C**) The observation of color change in the DTNB assay after 2 h of reaction without or with MCMSFT.

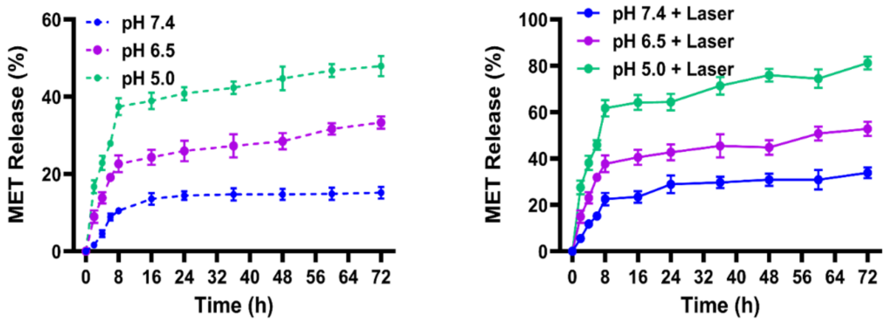

Fig. S5.

Responsive drug release of MCMSFT. Cumulative release curves of MET at different pH conditions without or with laser irradiation. The data are presented as the mean ± SD, n = 3.

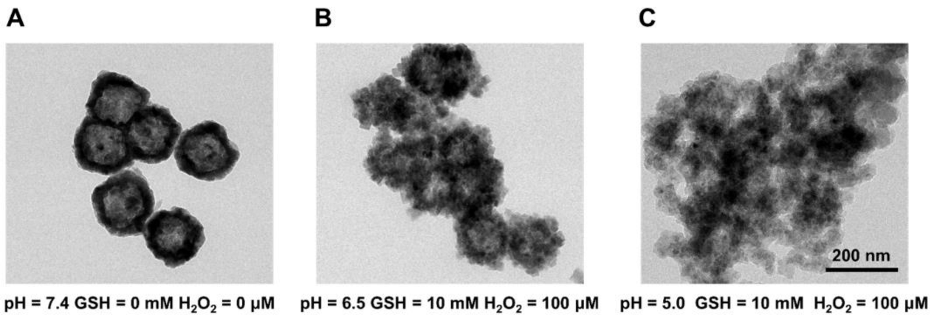

Fig. S6.

Biodegradation of MCMSFT. TEM images of MCMSFT after incubation with（**A**) physiological conditions, (**B**) tumor microenvironments, and (**C**) lysosome microenvironments mimicking buffers for 72 h.

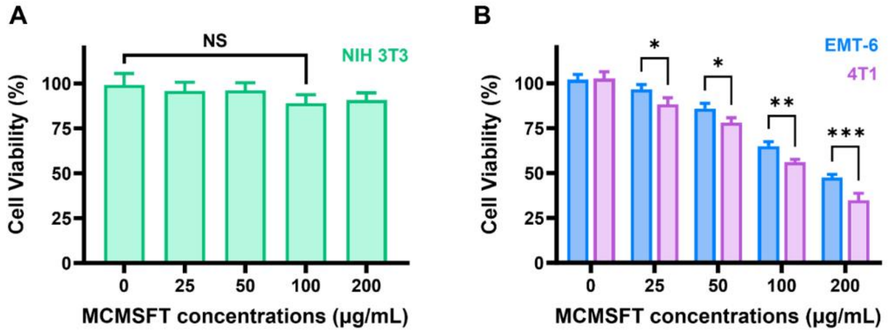

Fig. S7.

Cytotoxicity evaluation of MCMSFT. The cell viability of (**A**) NIH3T3 cells and (**B**) 4T1 and EMT-6 cells after incubation with various concentrations of MCMSFT for 24 h. The data are presented as the mean ± SD, n = 3, *$p < 0.05$, **$p < 0.01$, ***$p < 0.001$, NS: no significance difference.

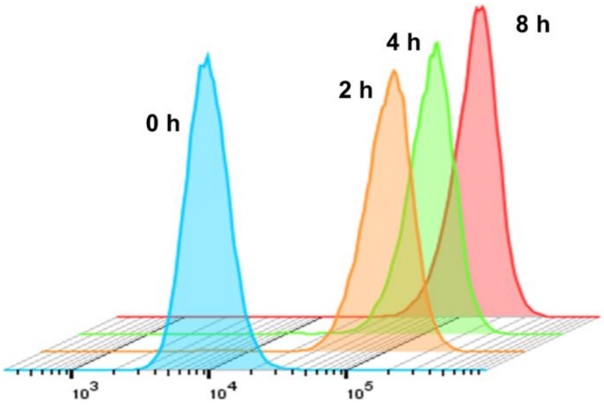

Fig. S8.

Cellular uptake of MCMSFT. The quantitative analysis of fluorescence intensity for cellular uptake of RhB-CMSFT for various time intervals.

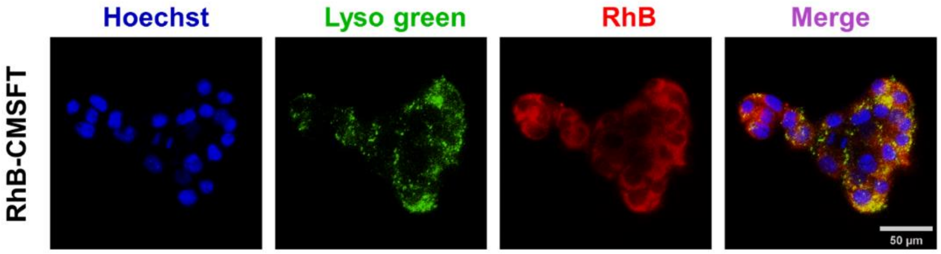

Fig. S9.

Subcellular localization of MCMSFT. Fluorescence images of 4T1 cells treated with RhB-CMSFT for 4 h. Lysosomes were labeled green with Lyso-tracker.

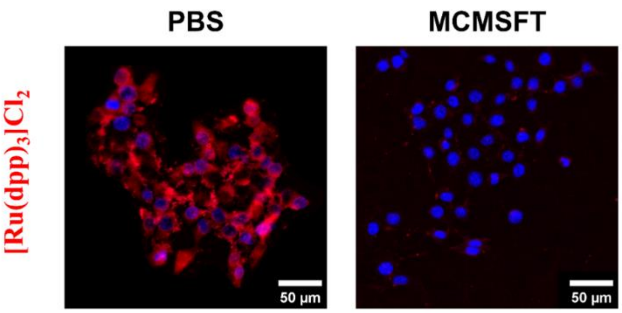

Fig. S10.

Hypoxia alleviation induced by MCMSFT. The intracellular O_2 generation examination for 4T1 cell after treatment with PBS or MCMSFT for 4 h. $[Ru(dpp)_3]Cl_2$ chosen as an intracellular oxygen indicator, with distinct red fluorescence under hypoxia while the red fluorescence is quenchable by oxygen molecules.

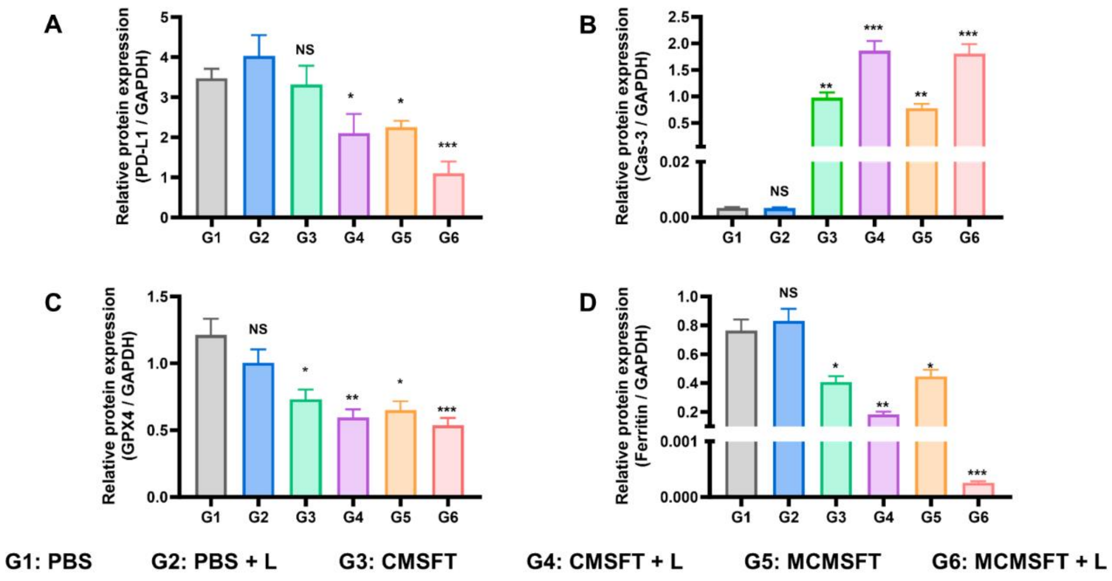

Fig. S11.

The protein expression quantitative analysis. The relative expression level of (**A**) PD-L1, (**B**) Cas-3, (**C**) GPX4, and (**D**) Ferritin in 4T1 cells after various treatments. The data are presented as the mean ± SD, n = 3, $*p < 0.05$, $**p < 0.01$, $***p < 0.001$, NS: no significance difference.

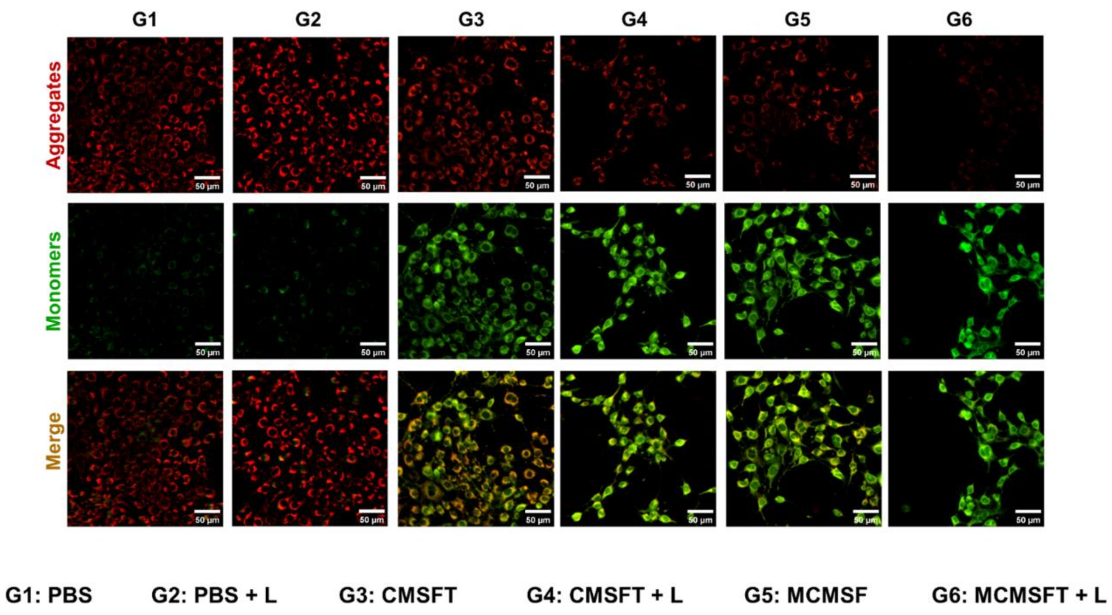

Fig. S12.

Mitochondrial damage assessment induced by MCMSFT. JC-1 staining of 4T1 cells after various treatment. The red JC-1 aggregates mean mitochondria with normal membrane potential, and the green JC-1 monomer indicates the mitochondria with depolarized membrane.

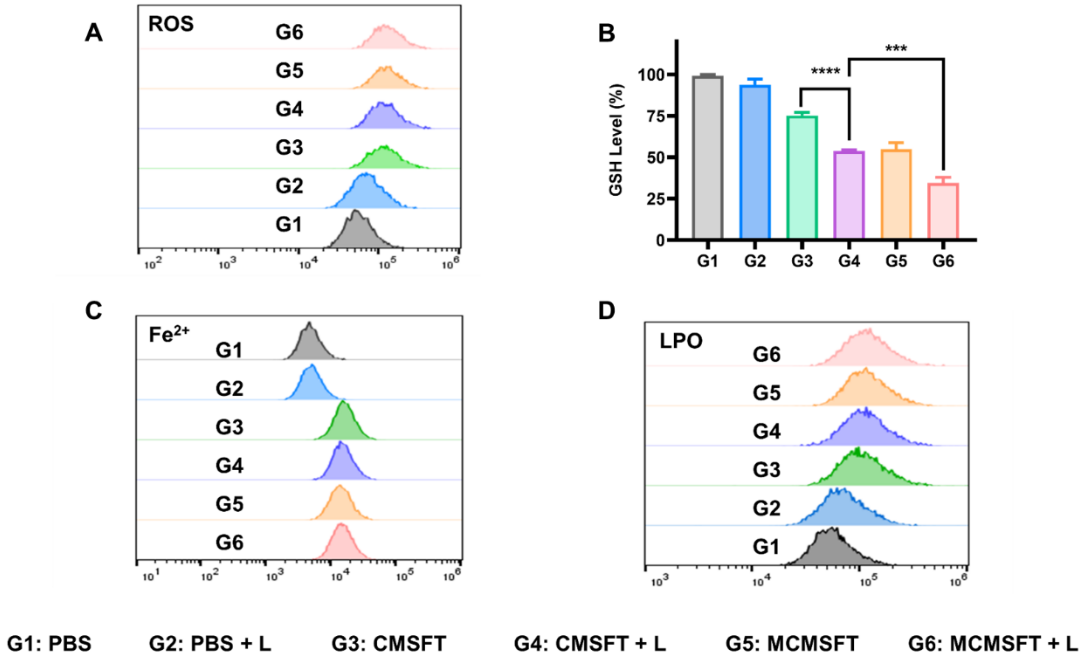

Fig. S13.

Quantitative analysis of mediators inducing ferroptosis. (**A**) the intracellular ROS, (**B**) the intracellular GSH, (**C**) the intracellular ferrous ions, and (**D**) the cellular LPO in 4T1 cells after various treatments. The data are presented as the mean ± SD, n = 3, ***$p < 0.001$, ****$p < 0.0001$.

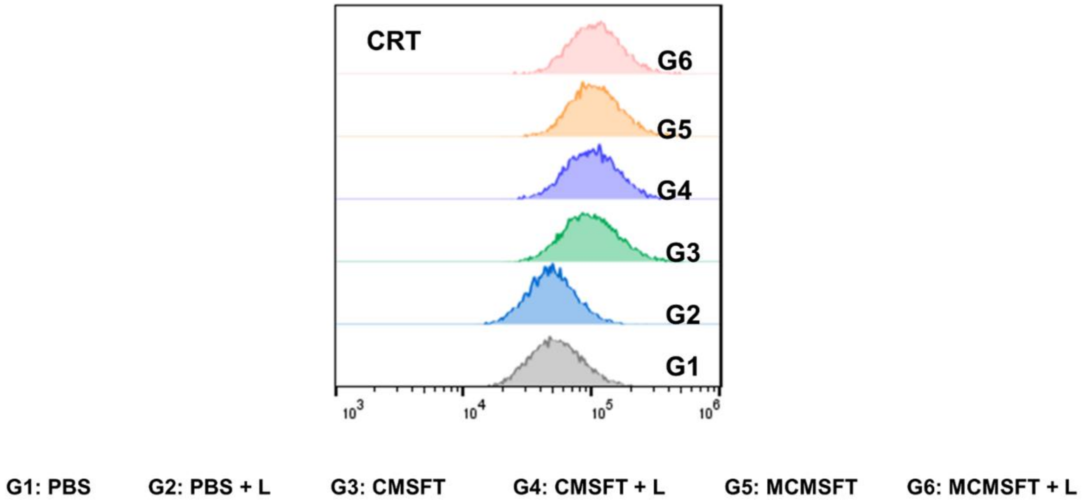

Fig. S14.

Flow cytometry quantitative analysis of CRT exposure. The fluorescence intensity of CRT in 4T1 cells after various treatments.

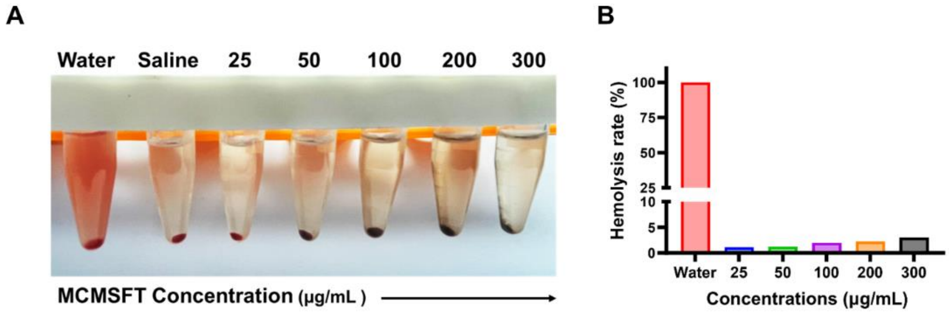

Fig. S15.

Biocompatibility evaluation of MCMSFT. (**A**) Hemolysis assay photograph and (**B**) hemolysis rate after treatment with different concentrations of MCMSFT for 2 h.

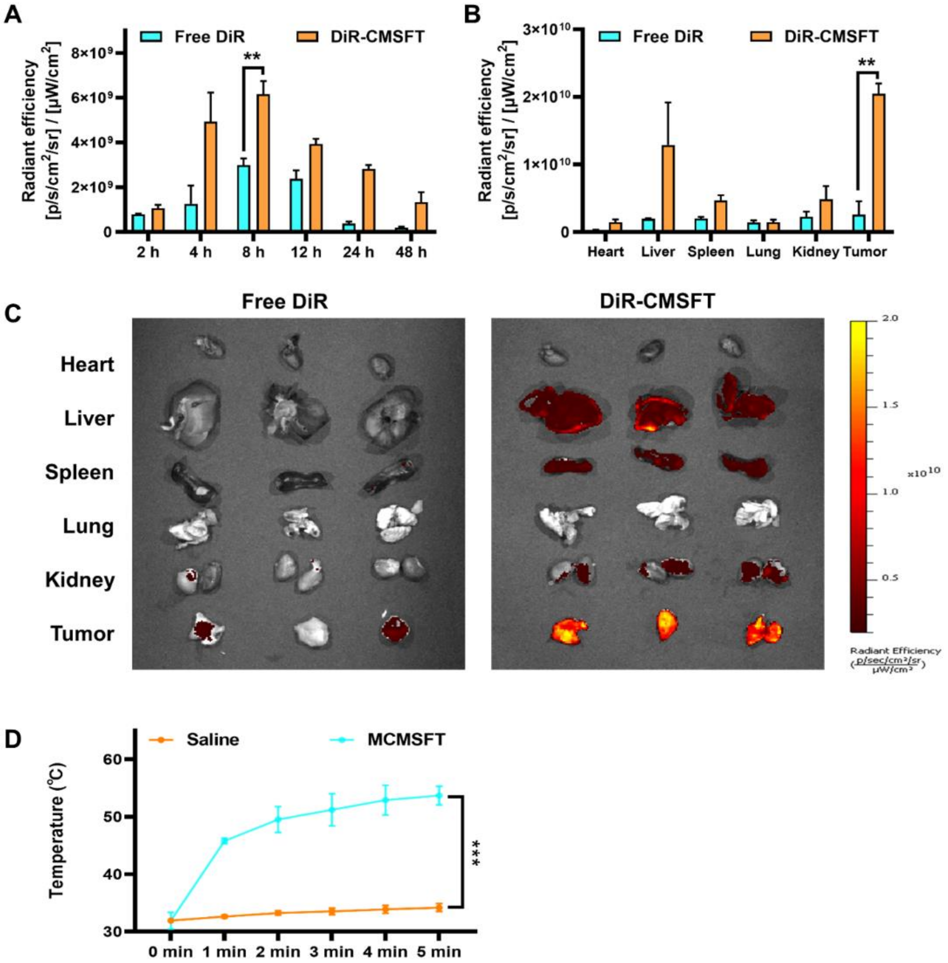

Fig. S16.

***In vivo* and *ex vivo* imaging analysis.** (**A**) Quantitative analysis of fluorescence intensity at tumor sites in living fluorescence imaging at different time points after post-injection of free DiR and DiR-CMSFT. (**B**) Quantitative analysis of fluorescence intensity and (**C**) Fluorescence images for *ex vivo* fluorescence imaging of isolated tumors and main organs from mice after 48 h post-injection of free DiR and DiR-CMSFT. (**D**) Quantitative analysis of temperature variation for *in vivo* photothermal imaging after 8 h post-injection of Saline and MCMSFT. The data are presented as the mean ± SD, n = 3, $^{**}p < 0.01$, $^{***}p < 0.001$.

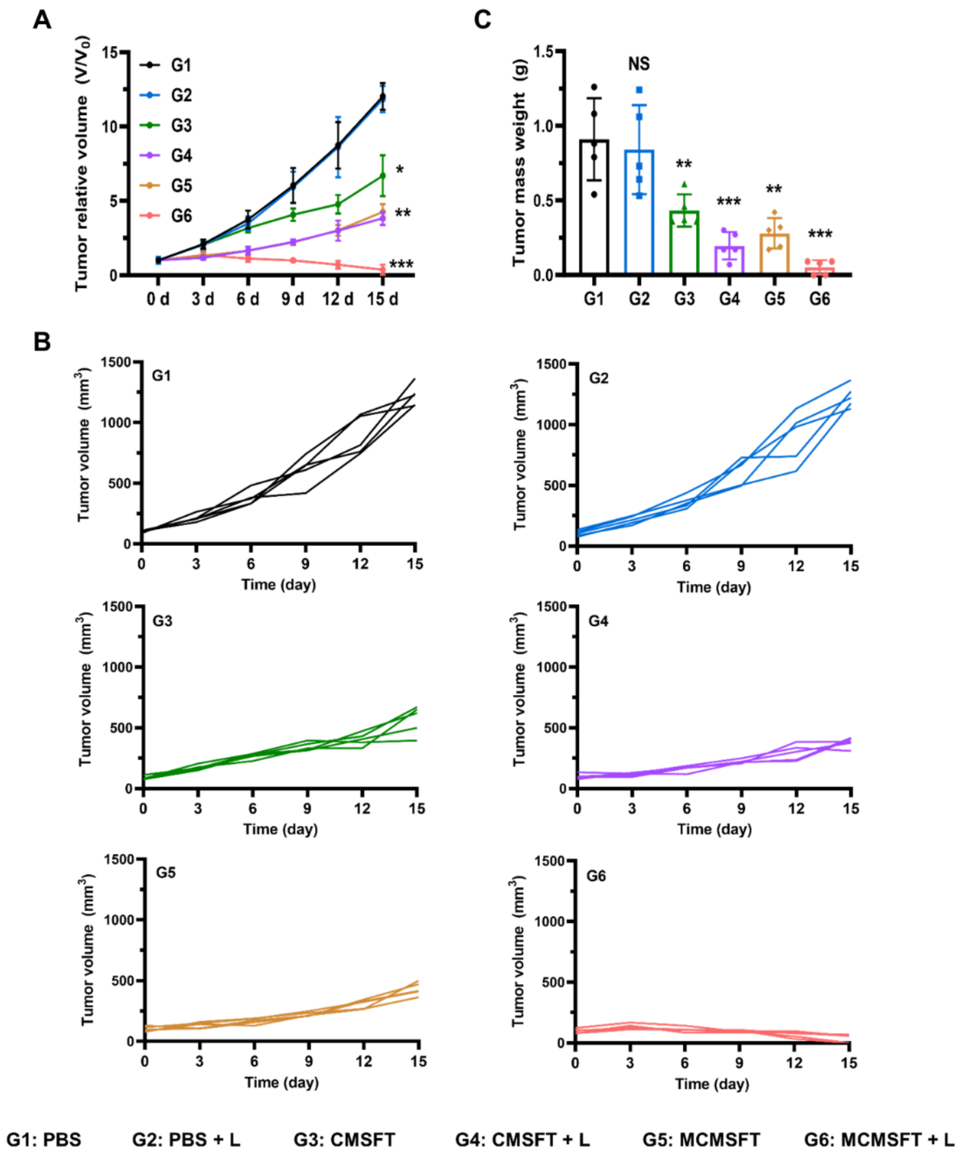

Fig. S17.

Quantitative analysis of tumor growth dynamics. (**A**) Average tumor growth curves. (**B**) Individual tumor growth curves during the therapeutic observation period. (**C)** Weights of tumors at the end of the therapeutic observation period. The data are presented as the mean ± SD, n = 5, *$p < 0.05$, **$p < 0.01$, ***$p < 0.001$, NS: no significance difference.

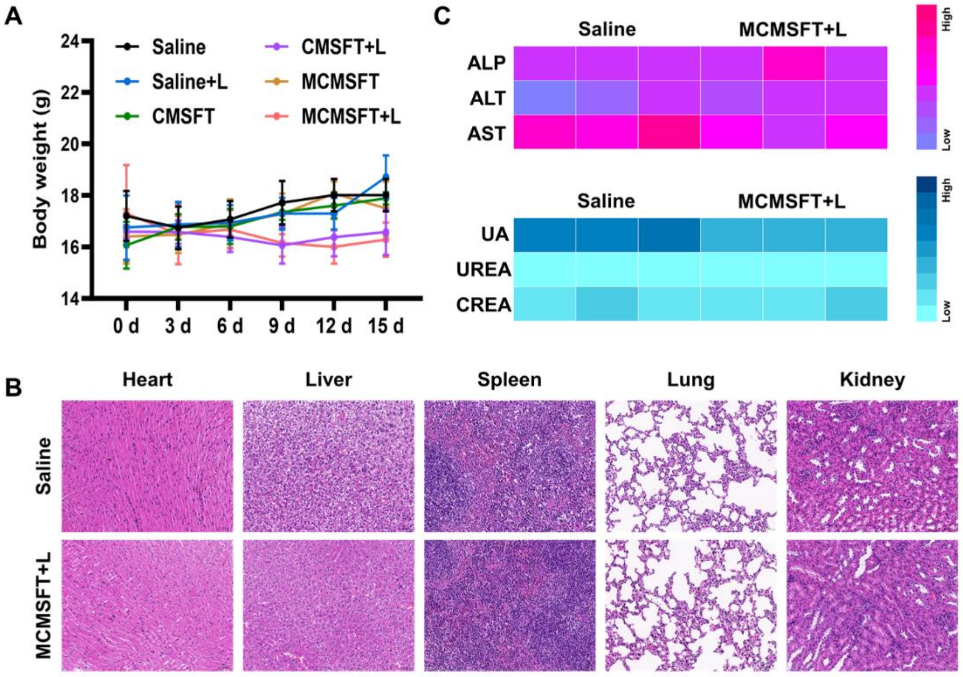

Fig. S18.

Biosafety evaluation of MCMSFT. (**A**) Body weights of mice during the therapeutic observation period. (**B**) Serum detection for biochemical indexes of liver and kidney functions after treatments. (**C**) Representative H&E staining images of the main organs after treatments. Scale bar: 200 μm. The data are presented as the mean ± SD, n = 5.

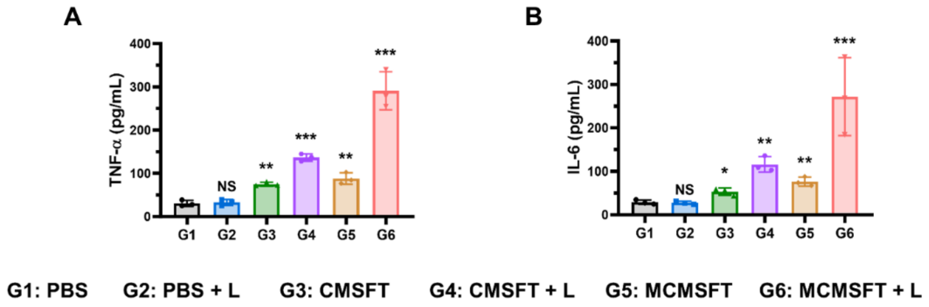

Fig. S19.

Serum cytokine detection. ELISA measurement of (**A**) TNF-α and (**B**) IL-6 levels in serum collected from mice after various treatments. The data are presented as the mean ± SD, n = 3, *$p < 0.05$, **$p < 0.01$, ***$p < 0.001$, NS: no significance difference.

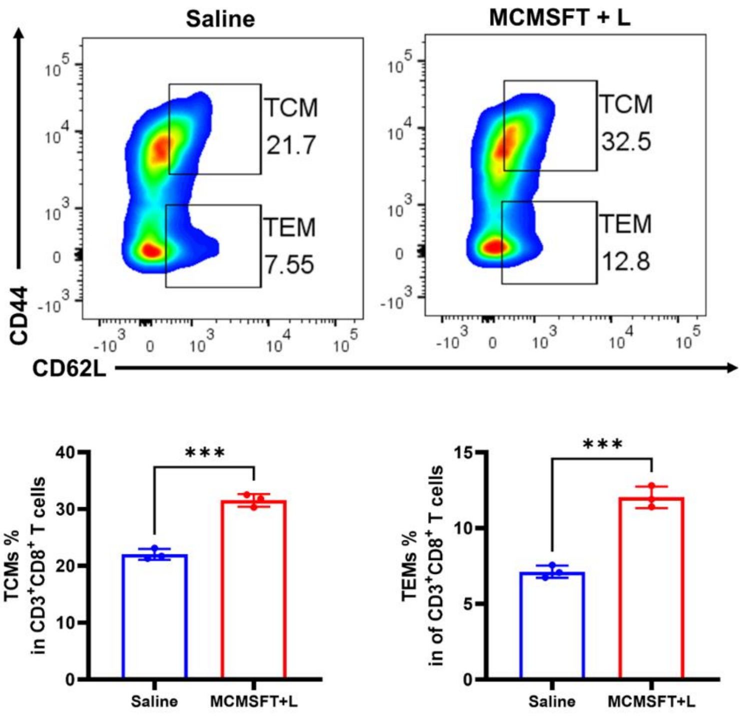

Fig. S20.

Immunological memory assessment. Representative flow cytometry plots and quantification analysis of central memory T cells ($CD3^{+}CD8^{+}CD44^{+}CD62L^{+}$) and effector memory T cells ($CD3^{+}CD8^{+}CD44^{+}CD62L^{-}$) in the spleen. The data are presented as the mean ± SD, n = 3, ***$p < 0.001$.
